# SFREEMAP - A simulation-free tool for stochastic mapping

**DOI:** 10.1186/s12859-017-1554-7

**Published:** 2017-02-22

**Authors:** Diego Pasqualin, Marcos Barbeitos, Fabiano Silva

**Affiliations:** 1 Departmento de Informática, Universidade Federal do Paraná, Caixa Postal 19081, Curitiba, PR 81531-980 Brazil; 2 Departmento de Zoologia, Universidade Federal do Paraná, Caixa Postal 19020, Curitiba, PR 81531-990 Brazil

**Keywords:** Phylogenetics, Ancestral states, Rate of evolution

## Abstract

**Background:**

Stochastic mapping is frequently used in comparative biology to simulate character evolution, enabling the probabilistic computation of statistics such as number of state transitions along a tree and distribution of states in its internal nodes. Common implementations rely on Continuous-time Markov Chain simulations whose parameters are difficult to adjust and subjected to inherent inaccuracy. Thus, researchers must run a large number of simulations in order to obtain adequate estimates. Although execution time tends to be relatively small when simulations are performed on a single tree assumed to be the “true” topology, it may become an issue if analyses are conducted on several trees, such as the ones that make up posterior distributions obtained via Bayesian phylogenetic inference. Working with such distributions is preferable to working with a single tree, for they allow the integration of phylogenetic uncertainty into parameter estimation. In such cases, detailed character mapping becomes less important than parameter integration across topologies. Here, we present an R-based implementation (SFREEMAP) of an analytical approach to obtain accurate, per-branch expectations of numbers of state transitions and dwelling times. We also introduce an intuitive way of visualizing the results by integrating over the posterior distribution and summarizing the parameters onto a target reference topology (such as a consensus or MAP tree) provided by the user.

**Results:**

We benchmarked SFREEMAP’s performance against *make.simmap*, a popular R-based implementation of stochastic mapping. SFREEMAP confirmed theoretical expectations outperforming *make.simmap* in every experiment and reducing computation time of relatively modest datasets from hours to minutes. We have also demonstrated that SFREEMAP returns estimates which were not only similar to the ones obtained by averaging across *make.simmap* mappings, but also more accurate, according to simulated data. We illustrate our visualization strategy using previously published data on the evolution of coloniality in scleractinian corals.

**Conclusion:**

SFREEMAP is an accurate and fast alternative to ancestral state reconstruction via simulation-based stochastic mapping.

**Electronic supplementary material:**

The online version of this article (doi:10.1186/s12859-017-1554-7) contains supplementary material, which is available to authorized users.

## Background

“Because evolutionary patterns and processes unroll over long time spans, their simulations has become an important ingredient of evolutionary research” [1].

Stochastic mapping (SM) [2] is a simulation-based, increasingly popular technique of ancestral-state reconstruction (ASR). Unlike maximum parsimony (MP) or maximum likelihood (ML) based reconstructions, stochastic mapping allows for changes to occur along branches (anagenesis) instead of assuming that they only occur when lineages split (speciate), a strictly cladogenetic (or punctuated *sensu* [3]) view of character evolution. MP- or ML-based ASRs imply that, at most, one single change in a character state will be recovered at any given node regardless of the length of the incident edge (i.e. the duration of the preceding lineage). With the advent of dated phylogenies, it has become evident that some of these edge lengths may actually span millions of years. Given such timescales and the fact that most lineages that have ever lived are now extinct, several cladogenetic events will be missed when one samples extant taxa (as in most molecular phylogenies) and even when extinct ones are included in the analysis, for the fossil record is all but guaranteed to be incomplete. Hence, even in the rare situation of exhaustive taxon sampling, there will be plenty of room left for missed state changes along phylogenies with deep splits. The bias introduced by the missing nodes is known as the node density effect (NDE) [4], a positive relationship between the number of nodes through which a lineage passes and the amount of estimated evolutionary change.

NDE is particularly pervasive in MP reconstructions because they do not factor branch lengths in their estimates. ML reconstructions are built on Markov models which assume that character evolution is a stochastic process, fully expressed as a matrix (Q) of instantaneous, pairwise rates of change between character states. These rates can be analytically integrated to probabilities of state change along a branch of known length. Markov models are based on a fundamental property: future states depend only on the present state and not on the sequence of events that preceded it. This model reflects the memory-less nature of stochastic processes, i.e. they tend to become independent of their initial conditions in time. This implies that state probabilities on a node will converge to their underlying equilibrium frequencies when the length of the incident branch tends to infinity [5]. Thus, inferred probabilities will become more evenly split among possible character states when focal nodes are closer to the root of a phylogeny with long internal branches. This somewhat compensates for NDE because reconstructions tend to become more ambiguous as the distance between the focal node and the phylogeny tips increases. This ambiguity reflects the uncertainty about the existence of missing nodes along ancient lineages. However, such uncertainty may be rather frustrating if the researcher is only interested in knowing, for instance, whether the underlying topology supports more transitions away from a state than towards it. This type of information is readily obtained via SM.

SM also has issues of its own. Unlike Bayesian phylogenetic analysis (e.g. [6–8]) there are no convergence diagnostics to assess how many simulations must be run in order to obtain a sufficient sample size. If not enough simulations are run, the posterior distribution will be undersampled. Otherwise, the analysis will waste computational resources. Given the ever increasing power of most personal computers, it seems wiser to err on the side of the second option because execution times tend to be small anyway. However, the other strong assumption embedded in ASR is that the chosen topology is the correct one, which is hardly, if ever, satisfied. This assumption can be relaxed by conducting ASR on a set of trees that represent the uncertainty inherent to the process of phylogenetic inference. This is normally done by running ASR on every tree in a posterior distribution, sampled via Metropolis-Coupled MCMC (MC^3^) (e.g. [2, 9]). Because the sizes of these distributions tend to be quite large, computation time may now become an issue. Additionally, one is faced with the challenge of how to summarize the results of such analyses. Several SM simulations must be conducted on each tree in order to reduce the stochastic error intrinsic to this approach. When applied to a posterior distribution of trees, this means tens of simulations per tree, being each one of its branches potentially replicated thousands of times. Conceivably, one could approximate a per-branch expectation of the character’s evolutionary history by averaging across different simulations conducted on a single branch and then integrating expectations for this branch across all trees. This is analogous to the popular “relaxed molecular clock” approach to divergence time estimation [7] because it effectively factors phylogenetic uncertainty into the posterior distribution of the variable of interest.

It has been demonstrated ([10]) that per-branch expectations of the number of transitions (away from a state) and dwelling times (the fraction of the branch length that a character is expected to have “spent” or dwelt on a given state) can be approximated analytically in execution times which are orders of magnitude faster than simulation based stochastic mapping. To our knowledge, this algorithm is only implemented in the library Bio++. Besides being restricted to molecular data, this (C++) implementation will likely have a much smaller user base among researchers interested in ASR than a package written in R, a high-level programming language tailored for statistical computing. Here, we introduce SFREEMAP (Simulation-FREE stochastic MAPping), an R-based implementation of the algorithm described in ([10]), specifically designed to allow fast ancestral character state reconstruction on thousands of phylogenetic trees. Additionally, our package offers an easy, intuitive way of creating synoptic charts of the results on any reference topology provided by the user.

## Implementation

R [11] is a multiplatform computer language that provides a high level environment for data analysis and plotting. It is a broadly used tool for phylogenetics [1] with dozens of related packages [12] written by researchers and developers around the globe. SFREEMAP has it’s interface written in R with parts of it’s core transcripted to C (with help from *Rcpp* [13] package), which can significantly improve performance on computing intensive calculations. The source code was made freely available under GPL [14] license and can be found on https://github.com/dpasqualin/sfreemap. The reference manual and vignettes are available as Additional files 1 and 2, please see [AF2 Ref. Manual] and [AF1 Vignettes].

## Results

### Performance evaluation

**Fig. 1:**
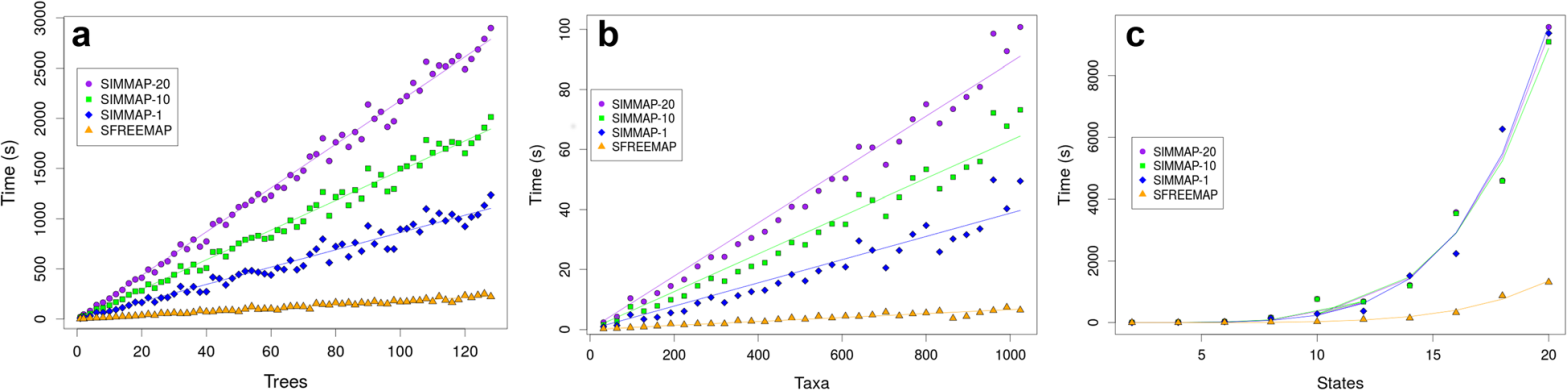
Benchmarking results for SFREEMAP. The curve labeled SIMMAP-1 was obtained using a single simulation, SIMMAP-10 using 10 simulations and SIMMAP-20 using 20 simulations. **a**) Increase in execution times with numbers of trees (256 terminals) during the mapping of a single 4-state character. Average performances were estimated from slopes (±SE), computed using R’s linear model (*lm* function), as seconds spent on each tree (SIMMAP-1 8.64 ± 0.11 s/tree, SIMMAP-10 14.80 ± 0.11 s/tree, SIMMAP-20 21.77 ± 0.12 s/tree, SFREEMAP 1.78 ± 0.02 s/tree). Intercepts were forced to 0 and determination coefficients (R2) exceeded 0.99 in all experiments. **b**) Increase in execution times with number of taxa for the reconstruction of a 4-state character on a single tree. Average performances were estimated from slopes (±SE) as seconds added per taxon (SIMMAP-1 0.038 ± 0.001 s/taxon, SIMMAP-10 0.063 ± 0.001 s/taxon, SIMMAP-20 0.088 ± 0.001 s/taxon, SFREEMAP 0.0066 ± 0.0002 s/taxon). Intercepts were forced to 0 and determination coefficients (R2) were over 0.97 in all experiments. **c**) Exponential increase in execution time with number of states. Experiments were run using one tree with 256 terminals. Lines were fit using the function *nls*, also implemented in R, assuming the formula y = bx^a^. We verified goodness-of-fit by adjusting a linear regression to log-log transformed data. R2 exceeded 0.89 in all experiments

We benchmarked the performance of SFREEMAP against an open source implementation of the simulation-based, stochastic mapping algorithm SIMMAP [15], available as the function *make.simmap* from the package *phytools* [16]. Experiments evaluated three main parameters thought to affect execution time: i) the number of trees, ii) the number of taxa and iii) the number of character states. Trees were always ultrametric and generated using *phytools*’ function *pbtree*. While evaluating one parameter, the others were held constant (defaults were 1 tree, 4 states and 256 terminals). Also, due to the stochastic nature of SIMMAP simulations, the researcher would want to perform several simulations (runs) to reduce the error inherent to the method. We employed 1, 10 and 20 simulations. *make.simmap* experiments were performed setting the parameter *Q* to “empirical”, meaning that a single *Q* matrix is estimated and subsequently used in all simulations. All experiments were conducted in a machine with 256GB of RAM memory, 32 cores processor Intel(R) Xeon(R) E5-4627 v2 running at 3.30GHz. Execution time increases linearly with the number of trees in all experiments (Fig. 1a). Due to the high determination coefficients of the regression lines (R^2^ > 0.99 in all cases), we can use slopes as accurate estimates of average performance, expressed as seconds spent on each tree (s/tree). In the case of SIMMAP-1 (i.e. *make.simmap* with a single simulation) the slope is 8.6 ± 0.11 s/tree. It took roughly 1.7× as long (14.8 ± 0.11 s) for 10 simulations (SIMMAP-10) and 2.5× (21.8 ± 0.12 s) for 20 simulations (SIMMAP- 20). Although execution times scale modestly with the number of simulations, SFREEMAP’s performance was far superior, approximately 1.8 ± 0.02 s/tree, or roughly 5× faster than SIMMAP-1, 10× faster than SIMMAP-10 and 12× faster than SIMMAP-20. Total execution time for the latter was almost 2900 s (≈48mins) for 128 trees, while SFREEMAP completed the same task under 4 mins (≈ 224 s). Usually, posterior distributions sampled by Bayesian phylogenetic analyses are made up by thousands to tens of thousands of trees. In order to obtain a new distribution made up of quasi-independent samples, they are normally sub-sampled (“thinned”) in order to break high temporal auto-correlations among parameters, characteristic of MCMC-based sampling [9]. Nevertheless, even after thinning, posterior distributions will typically retain hundreds of trees (e.g. [17]), meaning that it should take a couple of hours for *make.simmap* to reconstruct the evolution of a single 4-state character, even when a relatively small number of per-tree simulations is employed.

Algorithmic complexity was also O(n) with respect to the number of taxa (terminals), although it increased modestly with the addition of new taxa i.e., under 1 s/taxon in every experiment (Fig. 1b). Again, increasing number of simulations had a limited impact on execution time (1.6× for SIMMAP-10 and 2.2× for SIMMAP-20), but performance was substantially higher in the case of SFREEMAP, whose increase in execution time was, on average, only 7x10^−3^s per taxon, running 6× faster than SIMMAP-1 and 13× faster than SIMMAP-20 (Fig. 1b). Although the performance gain may appear small in absolute numbers, it is worth noting that a 4-fold increase in the number of terminals, from 256 (the default in the case of Fig. 1a) to 1024, means that execution time for one tree would go from 22.5 to 90 s in the case of SIMMAP-20. In contrast, the difference would be 5.9 s/tree (from 1.7 to 6.6 s) if the user chose SFREEMAP. Given the results for 128 trees with 256 terminals, SFREEMAP would finish in approximately 15 mins if the number of terminals were increased to 1024, whereas computation time would go from 48 mins to more than 3 h in the case of *make.simmap*.

The estimation of the *Q* matrix, a first step for both *make.simmap* and SFREEMAP, is a high complexity nonlinear optimization problem, implemented in the latter using the Quasi-Newton [18] method available through *nlminb* function from the R *stats* package, and only solvable in reasonable time due the low dimensionality of the matrix. Still, performance was most affected by the number of character states, as evidenced by the exponential increase in execution time depicted in Fig. 1c. This is highlighted by the nearly identical *make.simmap*’s curves corresponding to increasing numbers of simulations, whose effect is virtually obliterated by that critical first step in all experiments. Once again, SFREEMAP’s estimated performance was superior, although the difference was not as large as the previous experiments (3.5× with respect to SIMMAP-20 in the case of 4 states and 8× in the case of 10 states). The range of simulated states is not very realistic, for most biological characters would seldom have more than 10 states. A notable exception are proteins, whose state space may be as large as 20 amino acids. However, in this case, the researcher would probably start the analysis with a user-provided *Q* matrix, computed from empirically derived substitution matrices such as the ones in the BLOSUM or PAM series, thus reducing execution times considerably. At any rate, these results show that *Q* matrix estimation is the main performance bottleneck for both *make.simmap* and SFREEMAP. Optimization of *phytools*’ implementation of this step should yield the greatest performance gain for ASR algorithms built on that package.

Algorithm complexity can thus be generalized as O(*t*∙*n*∙*s* ^3^), where *t* is the number of trees, *n* is the number of terminals and *s* is the number of character states. Because a fixed number of steps is performed on each branch (and the number of branches is a linear function of the number of terminals), algorithm complexity increases linearly as more terminals and/or trees are added to the problem. The term s^3^ comes from the decomposition and multiplication of square matrices whose dimensions are set by the number of character states, as described in steps (i) and (ii) of Minin & Suchard's paper [10], on page 5. This formula excludes *Q* matrix estimation, the first step in the algorithm. As discussed above, performance is strongly limited by this single step as currently implemented in *phytools*, classified as a nonlinear optimization problem with exponential search space regarding the number of character states.

### Accuracy verification

SFREEMAP returns the expectation (approximated as the average) of the number of transitions and dwelling times on a given state along a branch. We evaluated the accuracy of the method by comparing these values to the numbers of transitions and dwelling times of a binary character with known (i.e. simulated) evolutionary history. An ultrametric tree with 128 terminals was generated via “pure-birth”, with exponentially distributed speciation times, using the function *pbtree* from *phytools*. Character evolution on this tree was simulated via *sim.history*, also part of *phytools*. Additionally, we evaluated the accuracy of the results obtained using *make.simmap* for the same simulated data. As *make.simmap* is based on stochastic simulations, the number of replicates for each simulation becomes another parameter of interest because the error inherent to the algorithm should decrease as replication increases. When the option *Q* = “mcmc” is set, *make.simmap* first samples a number (specified by the user) of *Q* matrices using MCMC and then generates one simulation of the character history for each sampled matrix. We ran simulations with varying numbers of replicates, from 100 to 2500.

**Fig. 2:**
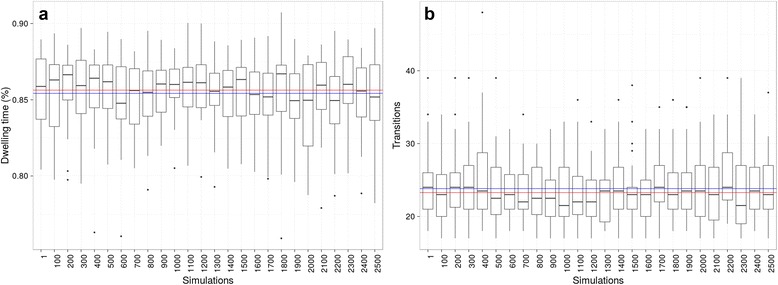
Comparison of SFREEMAP and *make.simmap* estimates. *make.simmap* estimates were obtained for stochastic mappings from a range of replicates, from 100 to 2500. Boxes correspond to standard deviations, dashes to means, whiskers to the min-max ranges and dots to outliers. **a**) Dwelling times, i.e. the fraction of the tree expected to have been “spent” on a given state, represented as percentages of tree length. **b**) Absolute numbers of transitions summed across tree branches

Figure 2a shows the tree-wide dwelling times for state 0. The boxplot summarizes *make.simmap* simulations results and the blue line is the overall mean dwelling time, computed across all these simulations. The red line corresponds to the grand mean of the per branch expectations, computed by SFREEMAP, which corresponds to the overall dwelling time on state 0 across the entire tree. Results for tree-wide numbers of transitions away from that state are shown in Fig. 2b. The blue line represents the overall mean obtained using *make.simmap* and the red line is the sum of the per branch expected number of transitions, computed by SFREEMAP.

**Fig. 3:**
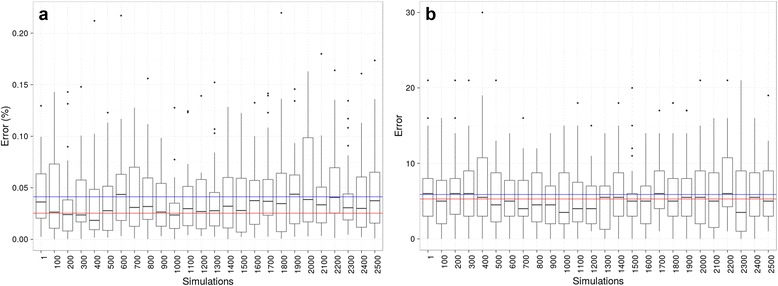
Accuracy verification. Absolute error for dwelling times and number of transitions. Errors were computed by comparing the differences between simulated (observed) and estimated dwelling times and absolute numbers of transitions across trees obtained using SFREEMAP and *make.simmap*. The latter were obtained for a range of replicates, from 100 to 2500. Boxes correspond to standard deviations, dashes to means, whiskers to the min-max ranges and dots to outliers. **a**) Dwelling times, i.e. the fraction of the tree expected to have been “spent” on a given state, represented as percentages of tree length. **b**) Absolute numbers of transitions summed across tree branches

Figure 3a and b represent the accuracy of SFREEMAP and *make.simmap* with respect to the simulated data. Accuracy was computed as the difference between estimated (computed as described in the previous paragraph) and observed (i.e. simulated) evolutionary trajectories. The blue and red lines in Fig. 3a represent the accuracy of dwelling-time estimation for *make.simmap* and SFREEMAP, respectively. The same color coding was used for the numbers of transitions in Fig. 3b. This experiment yielded three interesting results. The first is the closeness, on average, between estimates obtained using the *make.simmap* and SFREEMAP (represented by the blue and red lines, respectively (Fig. 2a and b). It suggests that the simulation-based approach can be replaced, with significant computational advantage, by the algorithm proposed in ([10]), whenever per-branch estimates will suffice. The second is that *make.simmap*’s stochastic error does not seem to taper off with increasing number of replicates. And finally, although both methods tend to overestimate dwelling times and numbers of transitions, SFREEMAP had, on average, greater accuracy than *make.simmap* (Fig. 2a and b). Nevertheless, virtually every set of *make.simmap* simulations had at least one replicate that recovered observed dwelling times and numbers of transitions with 100% accuracy (i.e. 0 error), although outliers were abundant in the case of numbers of transitions (Fig. 2b), highlighting the need to replicate the analysis in order to obtain more reliable estimates.

The results of this experiment should be taken with caution because observed data were obtained from a single simulation. Accuracy evaluation results could change if a larger number of empirical simulations under the same model and/or different parameters (i.e. numbers of character states, tree topology and branch lengths, etc.) were tested. The full evaluation of the algorithm’s accuracy is beyond the scope of this paper. The aim of this section is merely to demonstrate that the results generated by SFREEMAP agree with the theoretical expectations of its underlying algorithm.

### Graphical summarization

When working with a posterior of trees obtained via Bayesian phylogenetic analysis, it is desirable to summarize the probability distributions of the parameters of interest onto a target topology. For example, clade posterior probabilities may be mapped onto a maximum likelihood phylogeny obtained from the same data, or the distribution of divergence times may be represented as error bars centered on the internal nodes of a time-calibrated MAP (Maximum a Posteriori) tree. The latter approach is commonly used in studies focusing on Bayesian divergence times estimation and it is implemented, for instance, in the TreeAnnotator package, which is part of the BEAST suite [7].

**Fig. 4:**
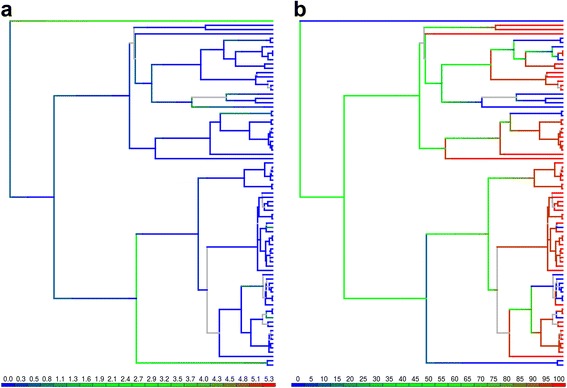
Example of graphical summarization of mapping results. Evolution of coloniality in scleractinian corals, data from [17]. In both cases, the MAP (maximum *a posteriori*) tree from a “thinned” posterior distribution of ultrametric trees (obtained using BEAST, *n* = 302) was used to summarize SFREEMAP’s results. The branch fraction painted with a given tone corresponds to the posterior probability of that tone. Gray branches are not present in other trees. **a**) Number of transitions from colonial to solitary states. Scale goes from 0 to over 5.34 transitions per branch. Branch coloring follows the same rationale as dwelling time. Note how greater number of transitions (represented by the “greenshift”) are observed on longer branches which, in the framework of stochastic mapping, offer more evolutionary opportunity for state change. **b**) Dwelling times in colonial state. Color scale goes from 0% (or 100% solitary) to 100% dwelling time on the colonial state. If 80% of a branch is painted pure red, this means that 80% of the trees in the posterior had equivalent branches expected to have been colonial throughout the entire duration of the corresponding lineage. If 80% of a branch is painted in pure green, then 80% of the equivalent branches were expected to have been colonial during half of the lineage duration. Note that coloring shifts to either red (colonial) or blue (solitary) towards the tips of the tree, matching the state observed on the terminals, whereas most of the internal branches closer to the root are green, due the uncertainty associated with increasing internal branch lengths

We introduce a graphical approach to summarize the results of simulation-free stochastic mapping conducted on several trees. The data in Fig. 4 were published in [17]. The tree corresponds to a MAP rRNA phylogeny of Scleractinian (hard) corals generated using BEAST. The binary character reconstructed onto each one of the 901 trees in the posterior is the presence or absence of coloniality (i.e. terminals are either colonial or solitary species). Given a branch, SFREEMAP returns the expected dwelling time on a state, expressed as a fraction of that branch’s length, and the absolute number of transitions away from that state. In order to summarize dwelling times (Fig. 4b), values are first normalized as percentages and converted into a corresponding color scale, whose tonality varies in 5% steps. Each branch in the target tree is then painted according to that scale, being the fraction covered by each tone proportional to the posterior probability of branches with corresponding dwelling times. For instance, (pure) red is used to represent 100% in the color scale. If 80% of the trees in the posterior have a branch *a* whose dwelling time on state 0 is 100%, then 80% of the total length of branch *a* in the target tree will be painted red. If the remaining 20% of the trees in the posterior had dwelling times on state 0 of 50% for branch *a*, the remaining 20% of branch a in the target tree will be painted green (Fig. 4b). A similar approach is adopted for the number of transitions, but in this case the color scale is adjusted between the 0 and the maximum number of transitions observed (Fig. 4a). If a branch in the target tree is not found in the other trees in the posterior, it will be colored gray.

## Conclusions

SFREEMAP provides a fast and accurate alternative to ancestral state reconstruction via simulation-based stochastic mapping. The package is specifically aimed at fast integration and intuitive representation of phylogenetic uncertainty associated with ASR. It does not return detailed estimates of the character’s evolutionary trajectory, which is needed, for instance, for character correlation analysis [2], as implemented in [15]. Nevertheless, dwelling times and number of transitions are important quantities for evolutionary biologists. The first allows the researcher to evaluate how prevalent a certain character state was during the evolution of a group. Quantifying numbers of transitions allows for testing of possible biases in character evolution (e.g. if coloniality evolved more often that it was lost during the history of hard corals). Besides being an useful quantity in itself, this latter variable may be converted to rates of evolution when normalized by branch lengths. Rate of evolution is a fundamental concept in biology, found inevitably at the core of virtually all hypotheses on the origin of genotypic, phenotypic and taxonomic biodiversity [19]. One major limitation of the current implementation in this regard is the fact that expectations correspond to transitions *away* from a state. Thus, this method does not allow direct computation of expected pairwise changes among states of non-binary characters (e.g. A → G or C → T in the case of DNA sequence data). Nevertheless, in cases where character states may be lumped into binary categories (such as transitions vs. transversions), it is possible to circumvent this limitation by estimating a *Q* matrix for this new (lumped) parameter space. Interested readers are referred to [10] for an example with synonymous vs. non-synonymous substitutions in HIV sequences.

Stochastic mapping is a powerful resource in the evolutionary toolbox, but we must guard against its unwarranted accuracy when a single topology is assumed to depict the “true tree”. The importance of accommodating uncertainty about the phylogenetic history of a group in comparative analysis is well established [20]. We believe this package provides an efficient way of accomplishing that within the framework of stochastic character mapping.

## Availability and requirements

**Project name:** Sfreemap

**Project home page:** https://github.com/dpasqualin/sfreemap

**Operating systems:** Platform independent

**Programming languages:** R

**License:** GNU GPL v3

## Additional files

Additional file 1: AF1 Vignettes. Sfreemap Vignettes. (PDF 367 kb)
Additional file 2: AF2 Ref. Manual. Sfreemap Reference manual. (PDF 119 kb)

## Abbreviations

ASR: Ancestral-state reconstruction
GPL: General Public License
MAP: Maximum *a posteriori*
MCMC: Markov chain Monte Carlo
MCMCMC/MC^3^: Metropolis-coupled Markov Chain Monte Carlo
ML: Maximum likelihood
MP: Maximum parsimony
NDE: Node density effect
SM: Stochastic mapping.
