# Supplementary material for: SFREEMAP - A simulation-free tool for stochastic mapping
Source: BMC Bioinformatics. 2017 Feb 22;18:123. doi: 10.1186/s12859-017-1554-7 (PMC5322606; doi:10.1186/s12859-017-1554-7)
Supplement: Additional file 1: — AF1 Vignettes. Sfreemap Vignettes. (PDF 367 kb) [file 12859_2017_1554_MOESM1_ESM.pdf]

Sfreemap

Diego Pasqualin, Marcos Barbeitos and Fabiano Silva

2016-12-18

Contents

1 Introduction **1**

2 Installing *sfreemap* **2**

3 New object classes **2**

4 Simple stochastic mapping **2**

4.1 Standard type 2

4.2 DNA type 5

5 Analysing mapped data with histograms **6**

5.1 Expected dwelling times for states of a branch across all trees 6

5.2 Expected dwelling times for states for all branches across all trees 9

5.3 Expected dwelling times for states for all branches on a group of trees 10

5.4 Expected number of transitions for states of one or more branches across all trees 11

5.5 Expected mutation rate for states of one or more branches across all trees 13

6 Analysing mapped data by plotting a tree **14**

7 Correlation matrix **20**

```
## Loading required package: ape

## Loading required package: sfreemap

##
## Attaching package: 'sfreemap'

## The following object is masked _by_ '.GlobalEnv':
##
##     sfreemap.corals.tips
```

1 Introduction

SFREEMAP is an analytical approach to obtain accurate, per-branch expectations of numbers of state transitions and dwelling times. We also introduce an intuitive way of visualizing the results by integrating over the posterior and summarizing the parameters onto a target reference topology (such as a consensus or MAP tree) provided by the user.

The following sections will guide you through installation and use of this tool.

2 Installing *sfreemap*

First make sure you have *libblas-dev* and *liblapack-dev* installed on your system.

To install the development version from github:

```
library(devtools)
install_github("dpasqualin/sfreemap")
```

The stable version can be installed from CRAN using:

```
install.packages(sfreemap)
```

Then, to load the package, use:

```
library("sfreemap")
```

3 New object classes

One new class of object extend existing data structure for phylogenetic trees: * **sfreemap:** complements "phylo" and "multiPhylo" classes from ape and phytools with *mapped.edge.lmt*, a matrix containing the expected value for the number of transitions among states.

4 Simple stochastic mapping

4.1 Standard type

The program accepts the parameter *type=standard* which should be used when the character is of morphological type. The dataset *sfreemap.corals.trees* and its corresponding tip values *sfreemap.corals.tips* can be used as example here.

Just to have an idea of the dataset we are working on, let's have a look at the first tree:

```
plot.phylo(sfreemap.corals.trees[[1]], cex=0.7)
```

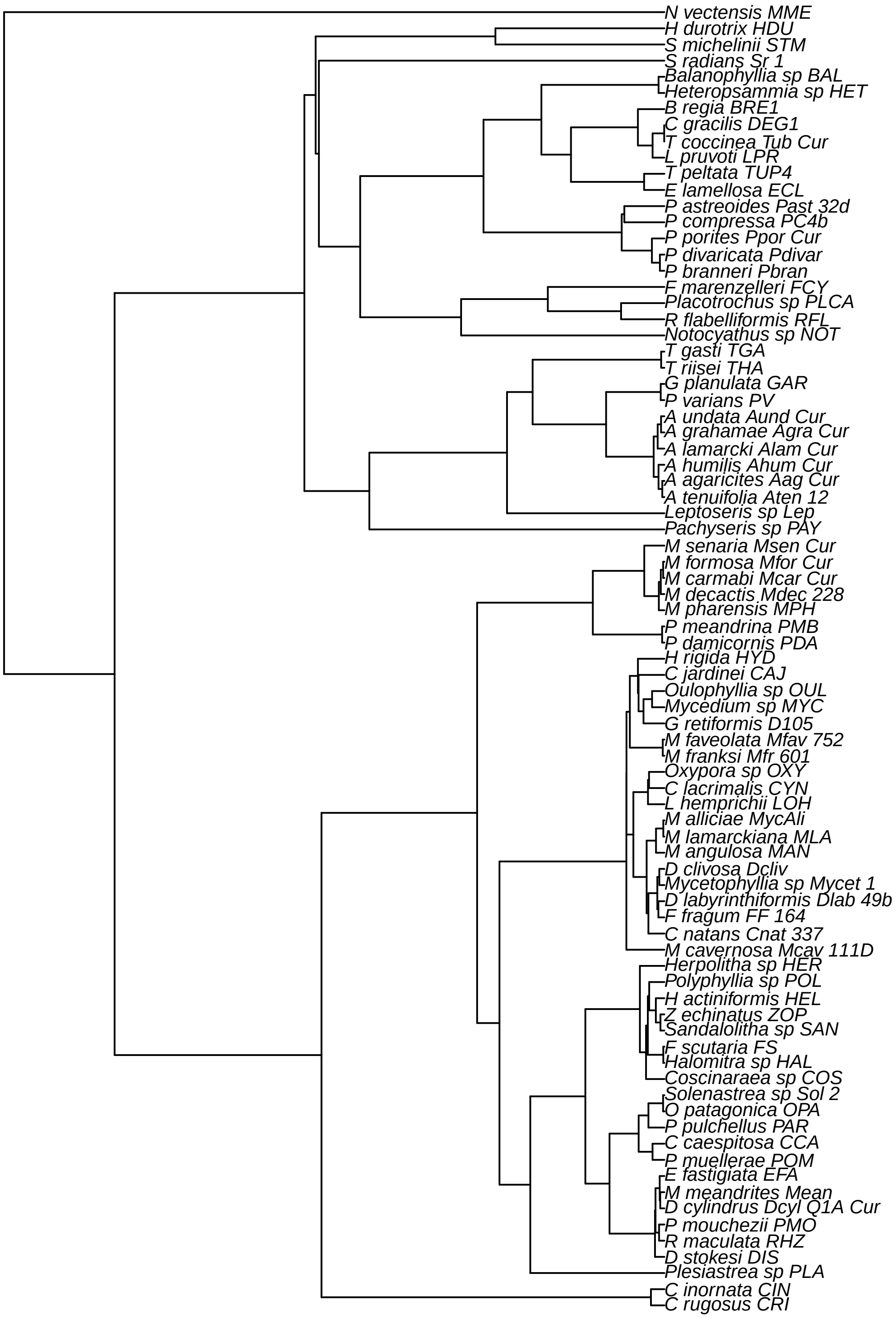

The command *sfreemap* runs by default with *method='empirical'* and *type='standard'*, so we don't have supply it now. Please check the package documentation for more details.

This function will run in parallel with a number of processes equal to the number of cores in your machine. If you want to disable parallel processing you can pass parallel=FALSE as a third parameter. Many other function of sfreemap presents this parameter.

```r
data <- sfreemap(sfreemap.corals.trees, sfreemap.corals.tips, parallel=FALSE)
class(data)
```

```
## [1] "sfreemap"   "multiPhylo"
```

```r
data
```

```
## 901 phylogenetic trees
```

All trees have the objects *mapped.edge* and *mapped.edge.lmt*, representing the expected dwelling times and expected number of transitions for each state on each branch of the tree. The following commands shows how to use these objects.

```r
t <- data[[1]] # Let's work with the first tree
t
```

```
##
## Phylogenetic tree with 81 tips and 80 internal nodes.
##
## Tip labels:
##  A_lamarcki_Alam_Cur, H_durotrix_HDU, C_jardinei_CAJ, B_regia_BRE1, L_hemprichii_LOH, A_agaricites_A
##
## Rooted; includes branch lengths.
```

```r
# Get the list of the first 10 edges
t$edge[1:10,]
```

```
##       [,1] [,2]
##  [1,]   82   83
##  [2,]   83   84
##  [3,]   84   85
##  [4,]   85   36
##  [5,]   85   48
##  [6,]   84   86
##  [7,]   86   87
##  [8,]   87   88
##  [9,]   88   51
## [10,]   88   89
```

```r
# Get the dwelling times for the first edge, the one connecting node 82 with 83
t$mapped.edge[1,]
```

```
## colonial solitary
##    39.77    44.47
```

```r
# Do the same thing using the branch name
t$mapped.edge['82,83',]
```

```
## colonial solitary
##    39.77    44.47
```

```
# Do the same thing for number of transitions
t$mapped.edge.lmt['82,83',]
```

```
## colonial,solitary solitary,colonial
##            0.2432            0.2161
```

4.2 DNA type

Use *type=dna* when working with nucleotides. The datasets *sfreemap.primates.trees* and *sfreemap.primates.dna.tips* can be used as example.

Again, we will run the program with only the first ten trees. The tips dataset has several characters, and again we are going to use just the first ten. What the program will do in this case is to run each tree against each character, in the order they appear in their respective objects. The result will be ten mapped trees. Same logic would apply for standard type.

```
sfreemap(sfreemap.primates.trees[1:10], sfreemap.primates.dna.tips[,1:10], parallel=FALSE)
```

```
## 10 phylogenetic trees
```

It is also possible to run the program passing a single tree as parameter and multiple characters. In this case we'll have ten trees as result, each one with a mapping that corresponds to a character.

```
sfreemap(sfreemap.primates.trees[[1]], sfreemap.primates.dna.tips[,1:10], parallel=FALSE)
```

```
## 10 phylogenetic trees
```

Of course, the user can also map a single character into multiple trees, like following:

```
res <- sfreemap(sfreemap.primates.trees[1:10], sfreemap.primates.dna.tips[,1], parallel=FALSE)
```

When using a single tree one can compute the mean value across trees for the number of transitions and dwelling times as follow:

```
# Using result from the last execution
mean.dt <- Reduce('+', lapply(res, function(x) x$mapped.edge)) / length(res)
mean.dt[1:10,] # just the first ten rows to give an idea..
```

```
##               -       a
## 60,61 6.013e-03 0.13635
## 61,62 2.616e-03 0.21027
## 62,63 6.944e-04 0.17085
## 63,64 2.599e-05 0.04004
## 64,65 3.463e-05 0.05752
## 65,36 1.301e-05 0.04059
## 65,66 1.202e-05 0.02736
## 66,37 4.384e-06 0.02853
## 66,38 2.289e-06 0.01787
## 64,18 1.713e-05 0.03332
```

Although the command above is quite typical for an R experienced programmer, it might not be as simple to remember and understand for a more regular user. Besides, what if we want the median instead of the mean? The command would be completely different. That is why we provide some tools to analyse the mappings produced by *sfreemap* command, which will be described in the next section.

5 Analysing mapped data with histograms

As we have shown, the object created by *sfreemap* can be analysed and manipulated to produce summaries and plots. In this section we will show some tools to make this task a lot easier.

For the examples we will map again the *sfreemap.corals.trees* dataset, but this time scaling the trees to have the same distance from the root to the terminals. This is important for analysing the number of transitions considering the same proportion on all trees.

```
trees <- rescale(sfreemap.corals.trees, height=1)
data <- sfreemap(trees, sfreemap.corals.tips, parallel=FALSE)
```

Now we will run the function below, which analysis mapping results on all trees, creating an object with the dwelling times and the number of transitions for every node of every tree. It tries to match nodes using function *matchNodes* from *phytools* packages, which consider two nodes to be the same when they share the same taxa. If you haven't rescaled the trees before but want to do it now, just pass on *scale.trees=1* (or any other value) to the function below.

```
base_tree <- data[[1]]
mpd <- map_posterior_distribution(base_tree, data, scale.branches=TRUE, parallel=FALSE)
```

The first parameter of the function is the so called "base tree", the tree on which the branches of the other trees will be compared to. Second parameter is all other trees to be analysed, *scale.branches* is used to represent the dwelling times regarding the percentage of time spent in the branch, instead of the absolute value (the expected number of transitions is not scaled, as it doesn't make sense to do so).

This function returns a list of three elements, each one representing a type of analysis.

1. *lmt* (labelled markov transitions): the expected value for number of transitions among states;
2. *emr* (expected markov reward): it's the dwelling times of states;
3. *mr* (mutation rate): the muration/evolution rate;

Each item on this list contains an array of three dimensions, *[x, y, z]*, where x indexes the trees, y indexes the states and z indexes the nodes. As an example, *mpd$emr[10,'colonial',83]* would return the dwelling times for state *colonial* on node *83* of the tenth tree.

If node *83* is not present on the tree number *10*, the value will be *NA*.

5.1 Expected dwelling times for states of a branch across all trees

Let's suppose we want to check the posterior distribution of states on a specific branch of the tree. Branches don't usually have names, so we will define a branch by it's ending node. For example, the code below will plot our base tree and it's correspondent node numbers.

```
plot.phylo(data[[1]], cex=0.7)
nodelabels(cex = .75, bg = "yellow")
```

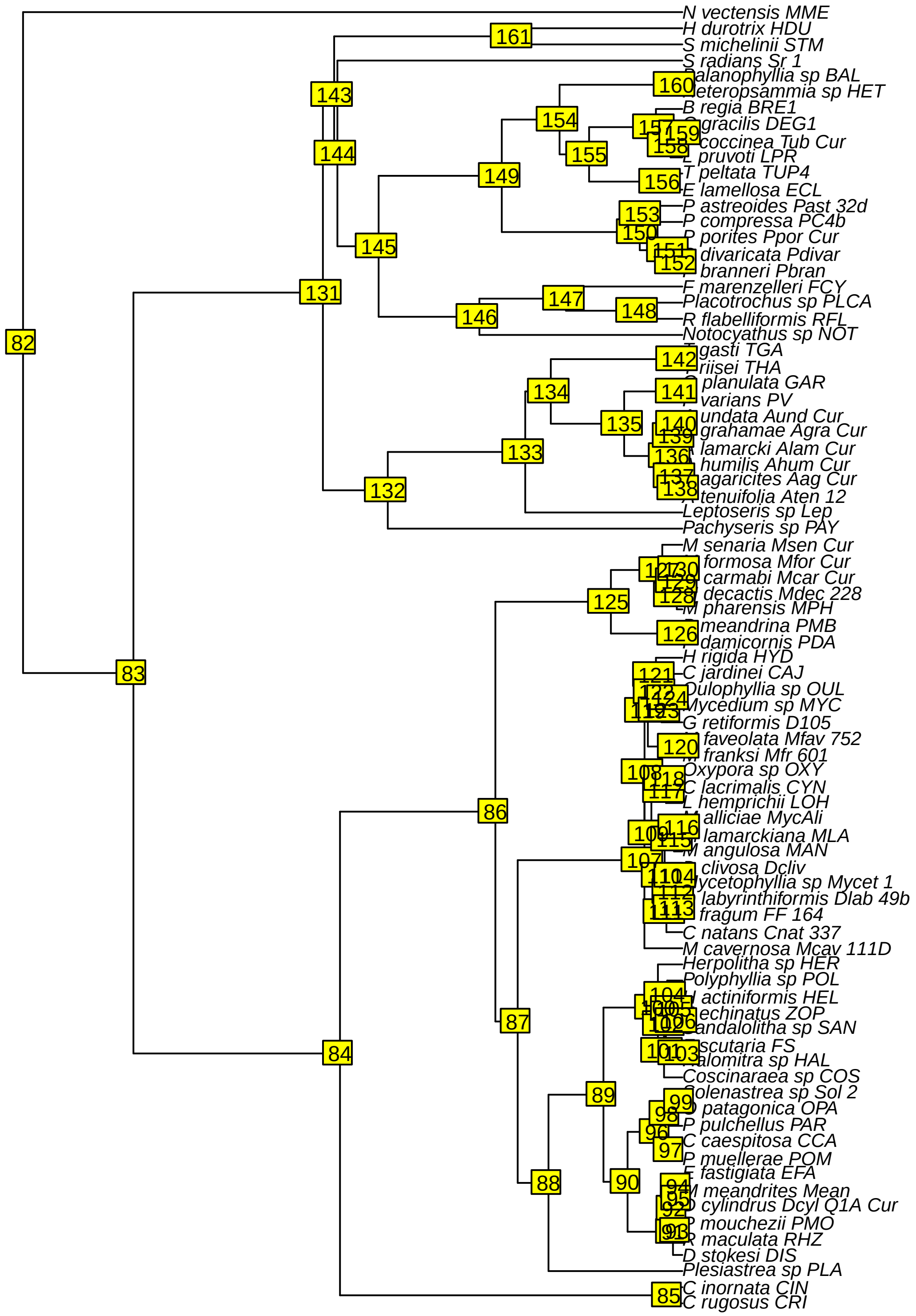

Picking one node, let's say *89*, we can see the posterior distribution for dwelling times on the branch that **ends** on node *89* by typing the following command (these are the default values for arguments *conf_level*, *number_of_ticks* and *type*, so you can omit it and get the same results):

```
plot_distribution_chart(mpd, 89, conf_level=95, number_of_ticks=20, type='emr')
```

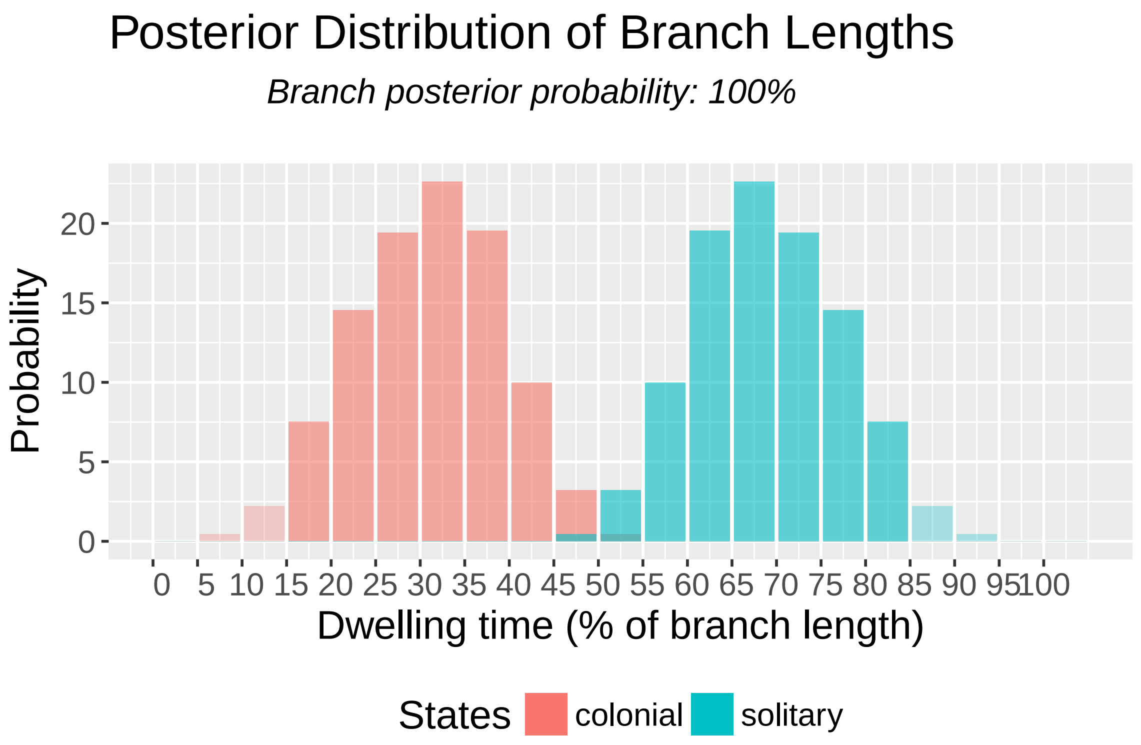

The interpretation is as follows: the *colonial* state was present with 95% certainty around 10% and 45% of the time on the branch ending on node 89 and, with equal certainty, the state *solitary* was present during 55% to 90% of the time on this branch. At the top of the chart, the text *branch posterior probability: 100%* means that this particular branch was present on 100% of the trees given as argument to the function *map_posterior_distribution* and compared to out *base tree*.

It is also possible to plot only one of the states by supplying the *states* argument.

```
plot_distribution_chart(mpd, 89, states='colonial')
```

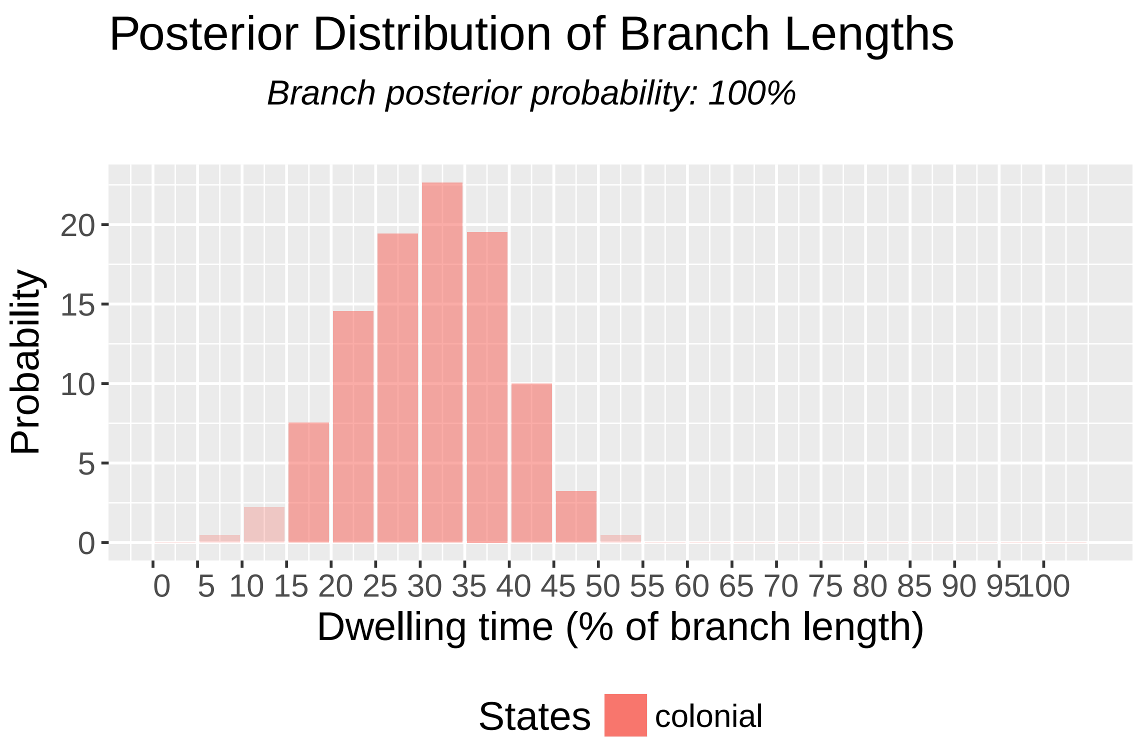

5.2 Expected dwelling times for states for all branches across all trees

Quite easy to do that, just omit the *node* parameter and the function will consider the distribution over all nodes.

```
plot_distribution_chart(mpd, type='emr')
```

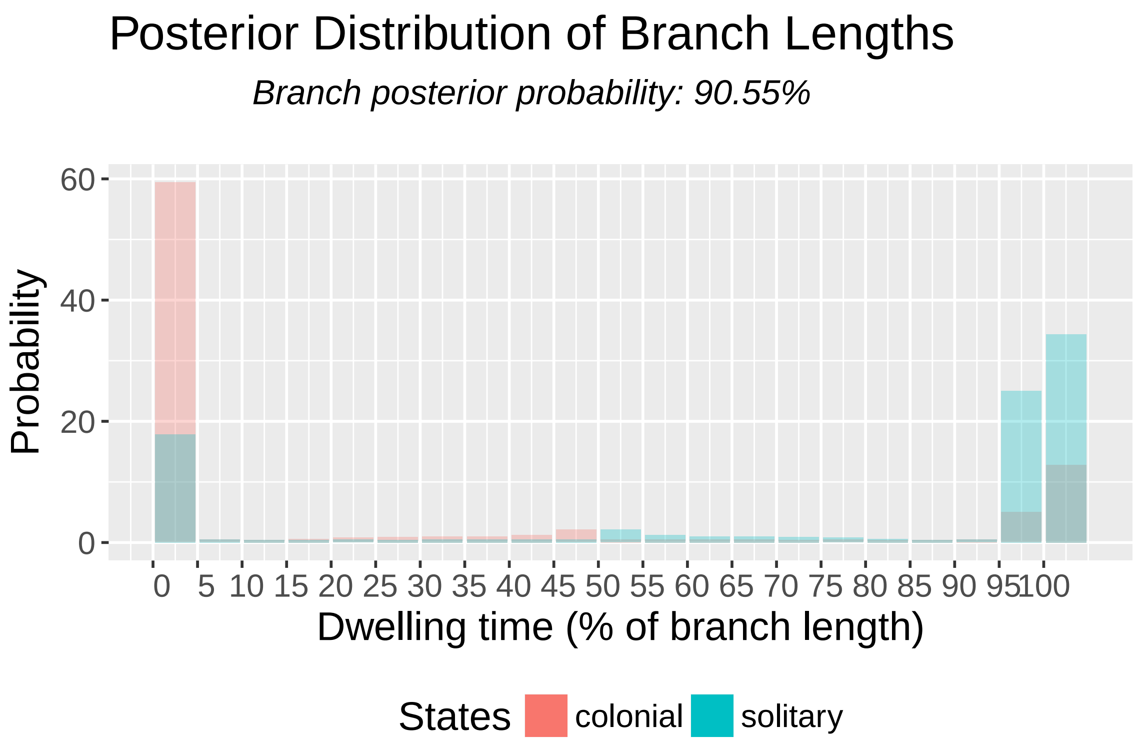

If you want to plot the distribution over a specific set of nodes just pass it as a vector, like this:

```
plot_distribution_chart(mpd, nodes=c(89,90), type='emr')
```

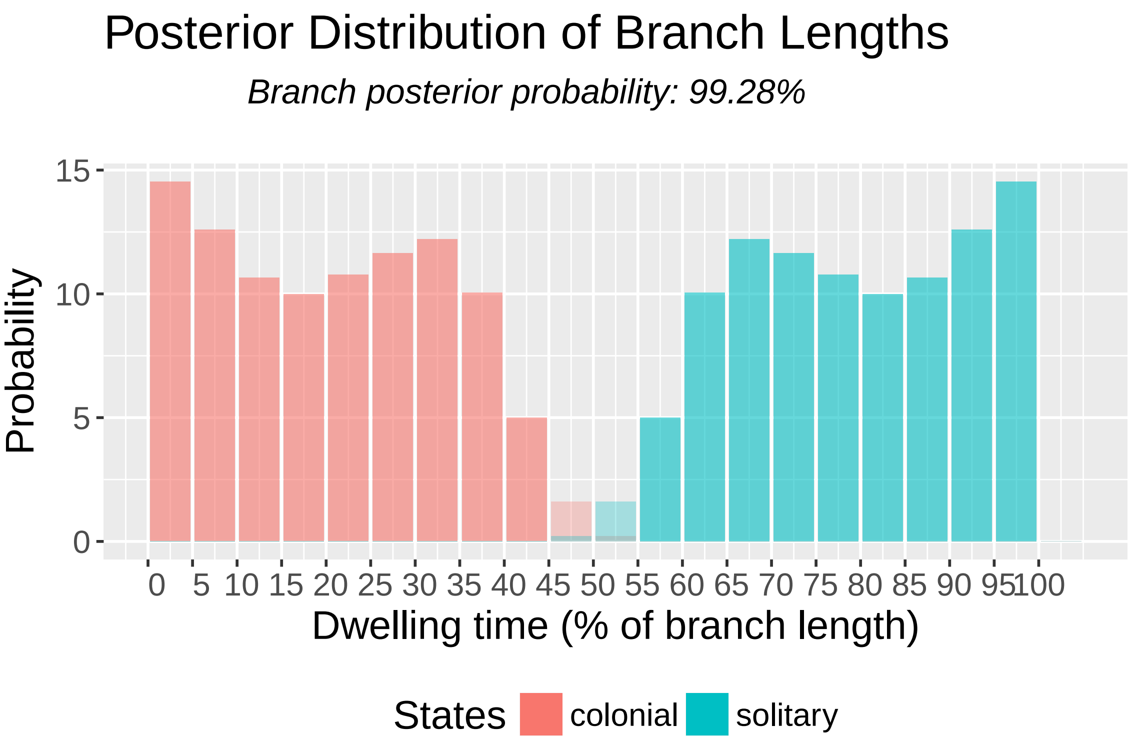

5.3 Expected dwelling times for states for all branches on a group of trees

To filter or limit the distribution over a specific group of trees, or maybe a single tree, pass on the argument *trees*, which work in the same way as *nodes* and *states*.

```
# get the odd trees
trees <- seq(1, length(sfreemap.corals.trees), 2)
plot_distribution_chart(mpd, nodes=89, trees=trees)
```

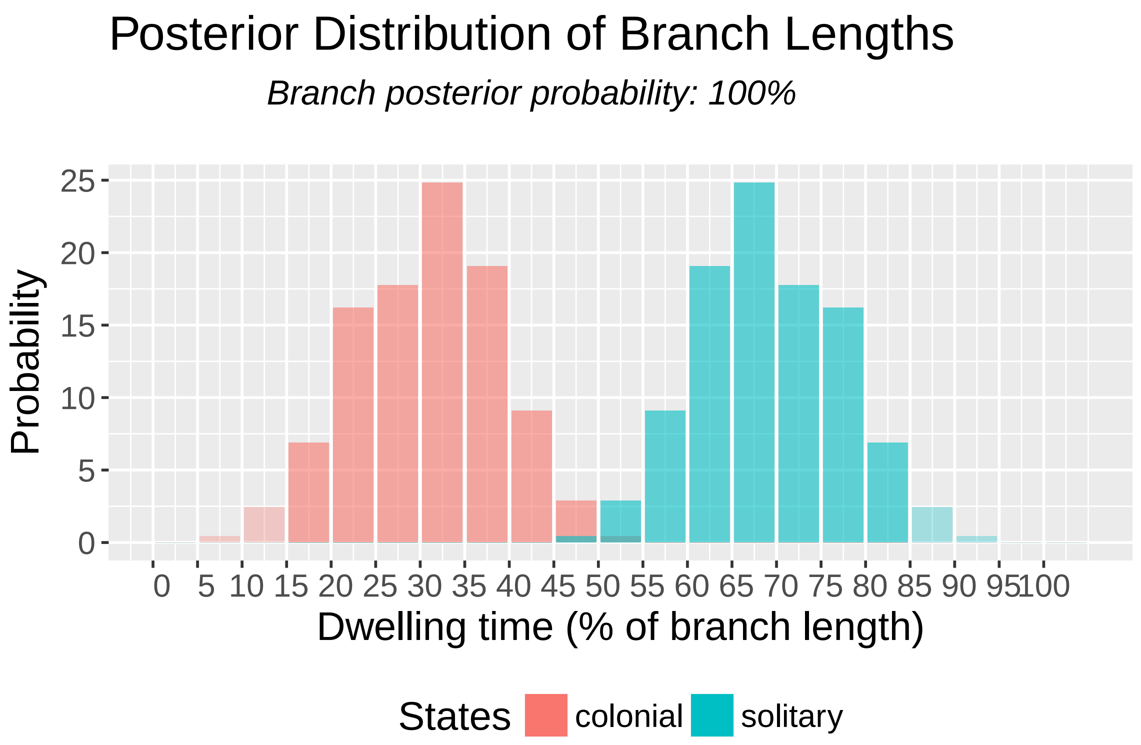

5.4 Expected number of transitions for states of one or more branches across all trees

Similar plots can be generated for the number of transitions using the same function and *mpd* object created before, just changing the parameter *type* from *emr* to *lmt* (labelled markov transitions).

As stated before, it doesn't make sense to scale the number of transitions according to the branch length, so here the absolute values are represented in the x-axis.

```
plot_distribution_chart(mpd, 85, type='lmt')
```

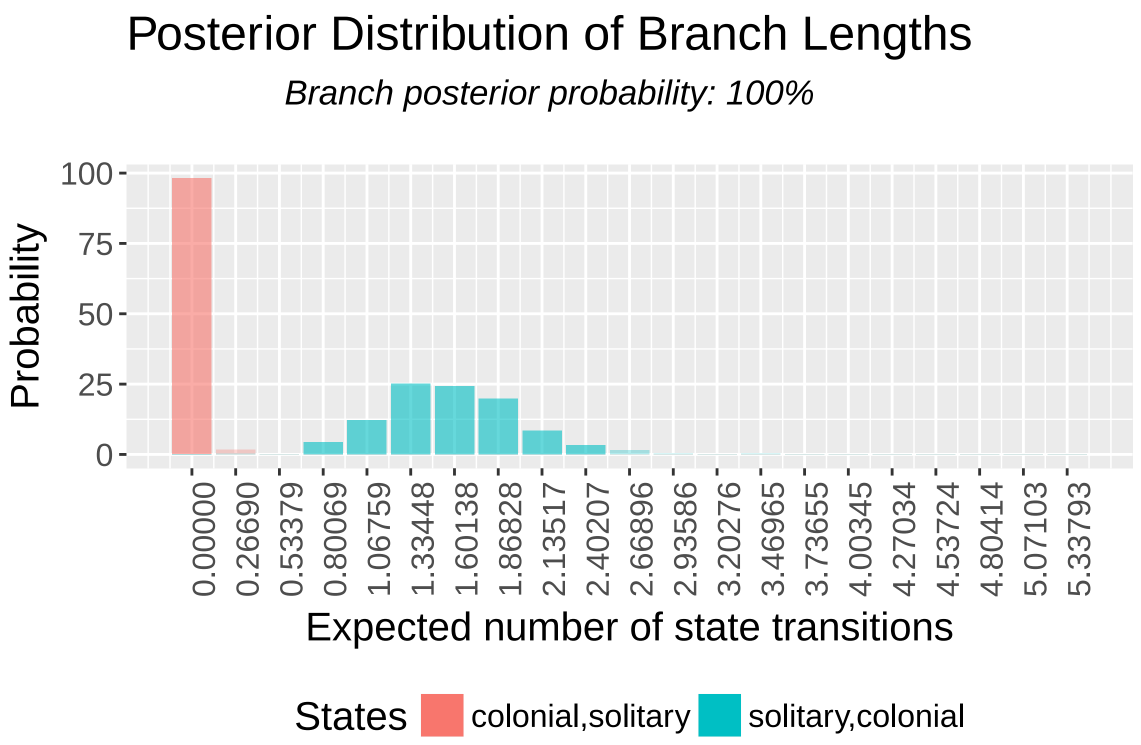

Needless to say, but you can plot a single state transition here:

```
plot_distribution_chart(mpd, 85, states='solitary,colonial', type='lmt')
```

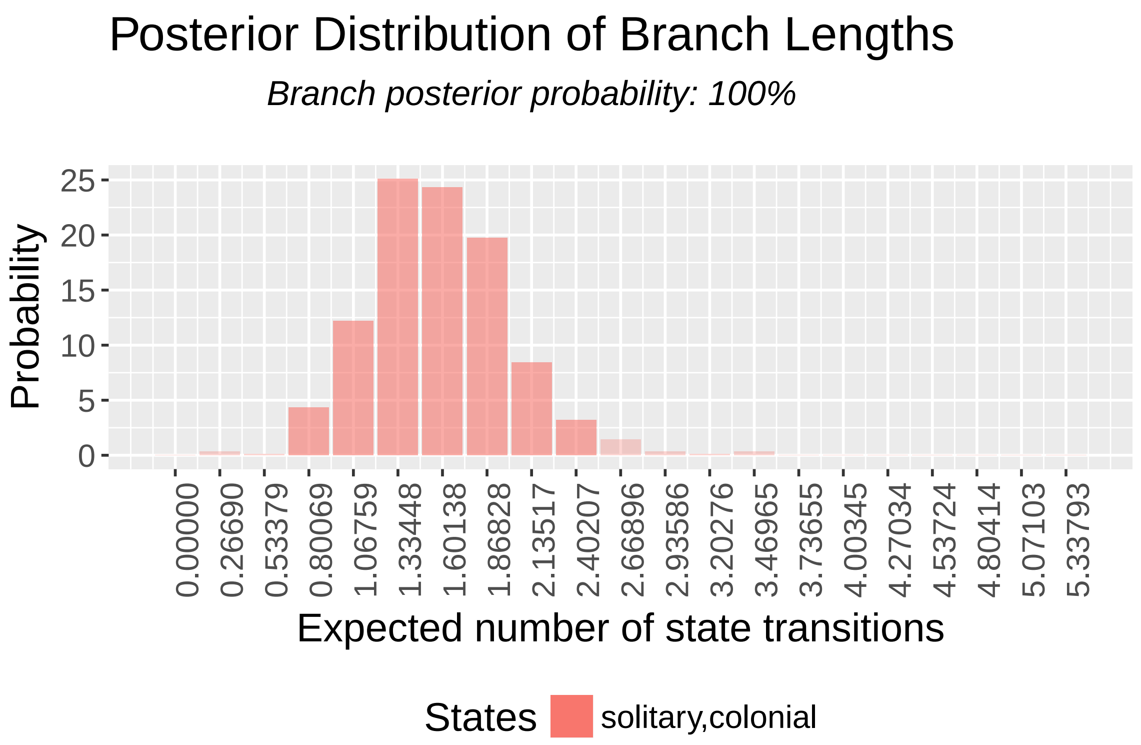

Example considering all nodes and all states:

```r
plot_distribution_chart(mpd, type='lmt')
```

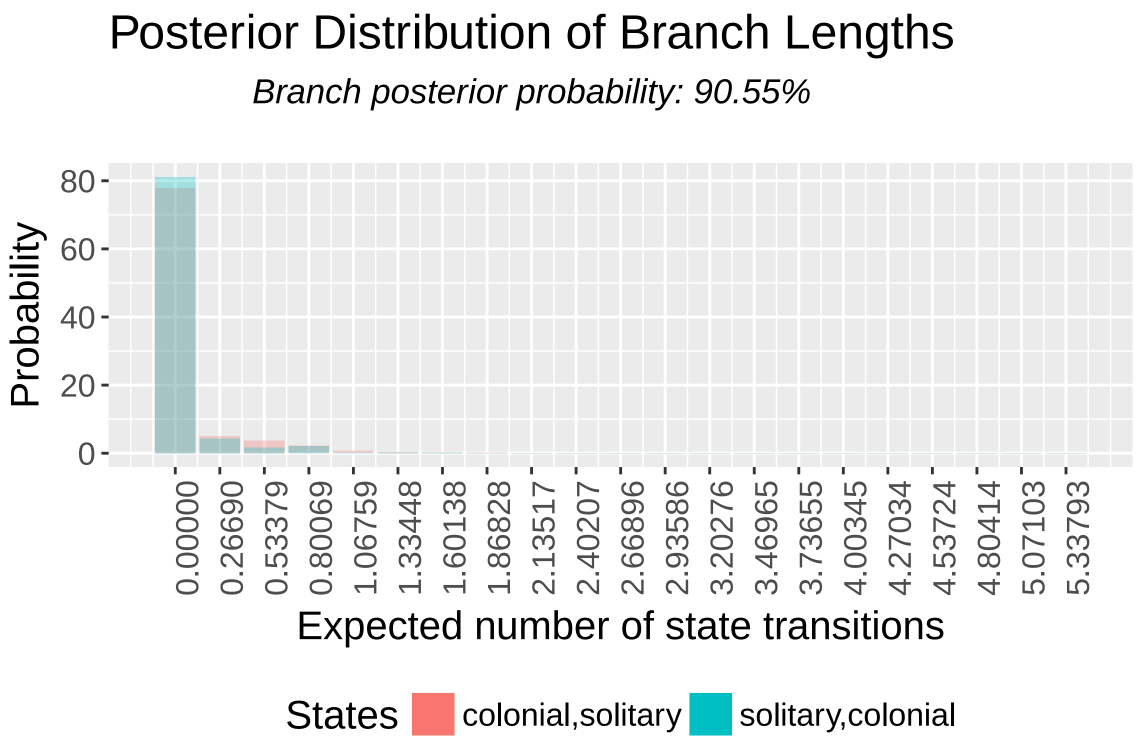

5.5 Expected mutation rate for states of one or more branches across all trees

As the last type of distribution plot, *mr (mutation rate)* shows the rate of mutation for states per branch. Parameters are very similar than before. It is possible to filter by trees, nodes and/or states.

Just as a reminder, it makes more sense to analyse mutation rate on trees where the distance between any leaf and it's root is equal. If the trees passed to *sfreemap* where not scaled in that way, it's possible to do that using the *map_posterior_distribution* function.

```r
base_tree <- data[[1]]
mpd <- map_posterior_distribution(base_tree, data, scale.trees=1, parallel=FALSE)
```

Now that we know that all values are scaled in a way that all leaf nodes are distant from the root node by *1* unit, we can then plot the distribution chart.

We can get the mutation rate for all states in a single tree (let's say, tree *2*) with the command below:

```r
mpd$mr[2,,85]
```

```
## colonial,solitary solitary,colonial
##         2.418e-05         5.955e-03
```

The mean mutation rate considering all trees and all states would be as simple as:

```r
apply(mpd$mr[,,85], 2, mean)
```

```
## colonial,solitary solitary,colonial
##         0.0002573         0.0058513
```

But it might be more interesting to have it plotted as a distribution:

```r
plot_distribution_chart(mpd, 85, type='mr')
```

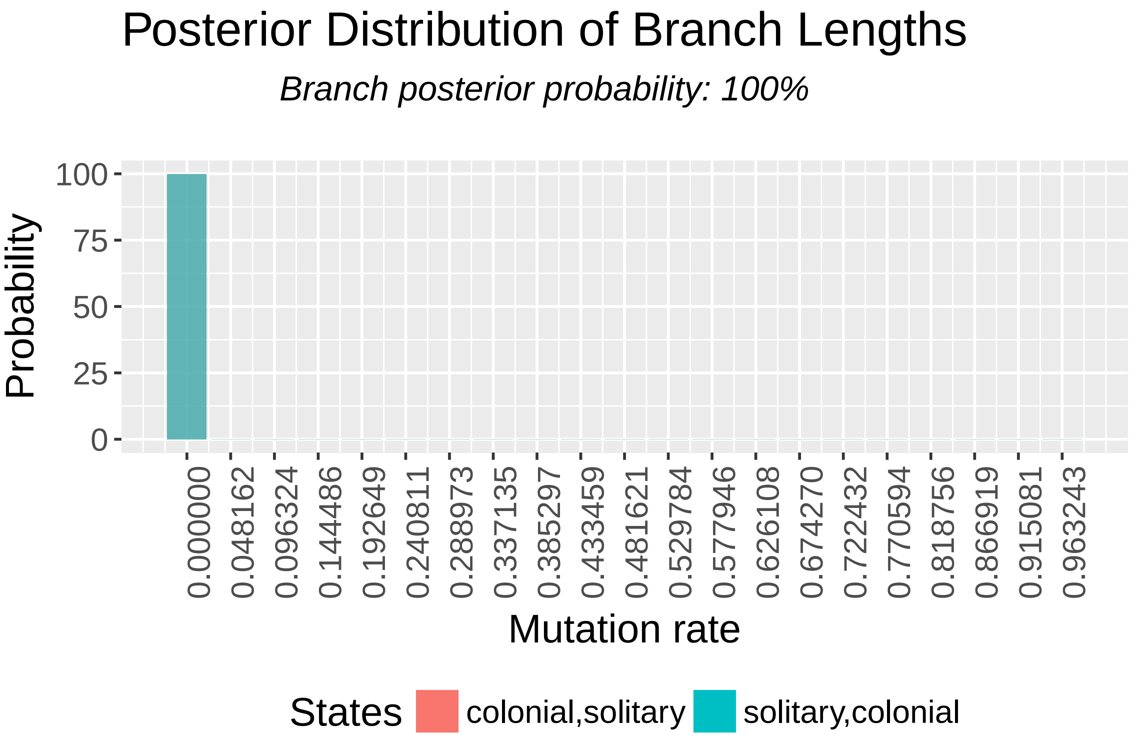

6 Analysing mapped data by plotting a tree

Sfreemap can show up mutation rate and dwelling times in a graphical representation of a tree. Fist let's generate a mapping for all trees of *sfreemap.corals.trees* dataset, and then map the postero distribution (as we did before).

```r
data <- sfreemap(sfreemap.corals.trees, sfreemap.corals.tips, method='empirical', parallel=FALSE)
mpd <- map_posterior_distribution(data[[1]], data, parallel=FALSE)
```

Now we can use the function below to plot a tree showing the distribution of time spent by a particular state (second argument). As the legend says, red shifted colors indicates that the state was more present, and on the other side of the spectrum, blue indicates less time spent for the state on that particular branch.

```r
plot_distribution_tree(mpd, 'colonial', type='emr', conf_level=95)
```

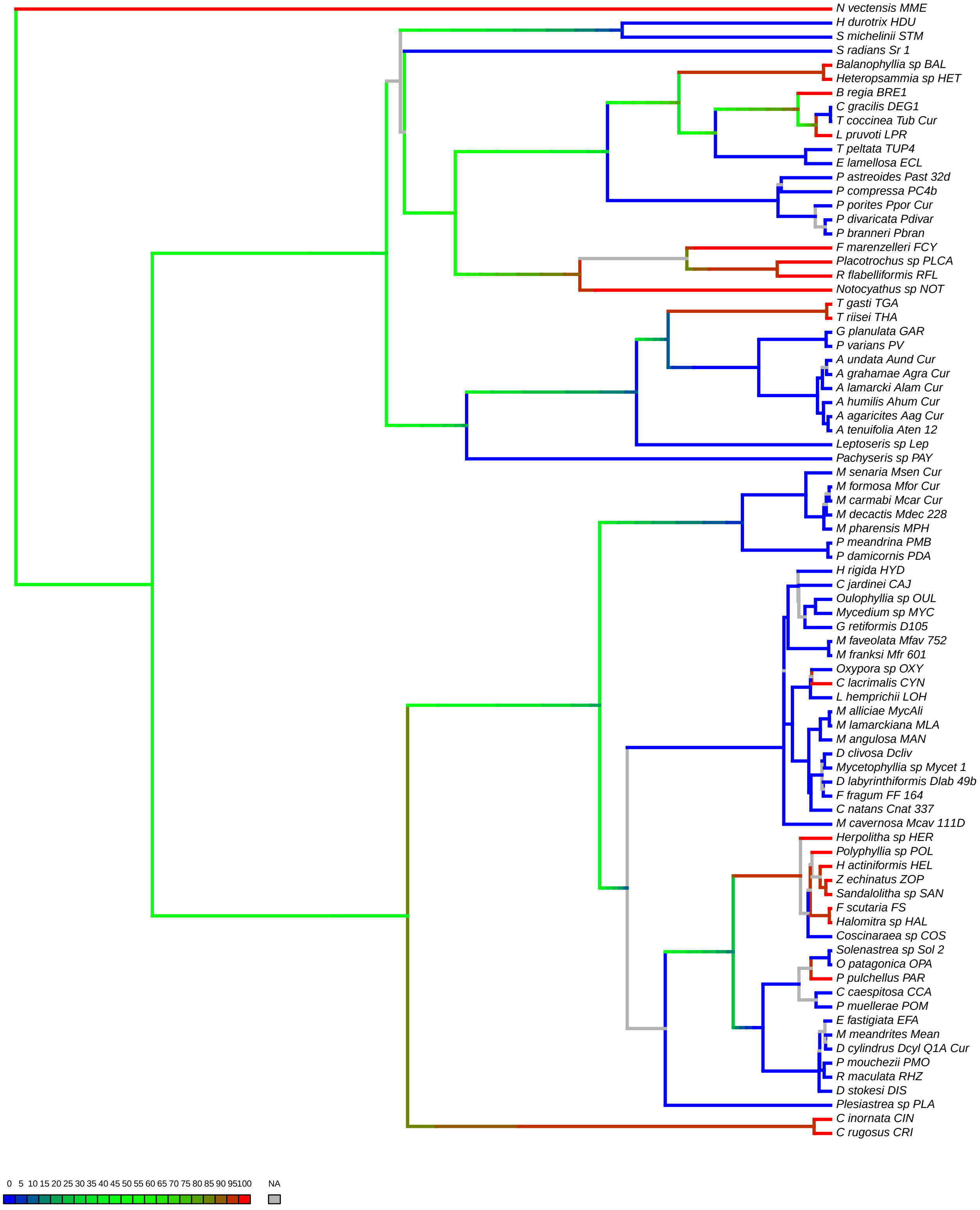


```
##
## Phylogenetic tree with 81 tips and 80 internal nodes.
##
## Tip labels:
##  A_lamarcki_Alam_Cur, H_durotrix_HDU, C_jardinei_CAJ, B_regia_BRE1, L_hemprichii_LOH, A_agaricites_Aa
```

```
##
## Rooted; includes branch lengths.
```

Now the same for the other state:

```
plot_distribution_tree(mpd, 'solitary', type='emr', conf_level=95)
```

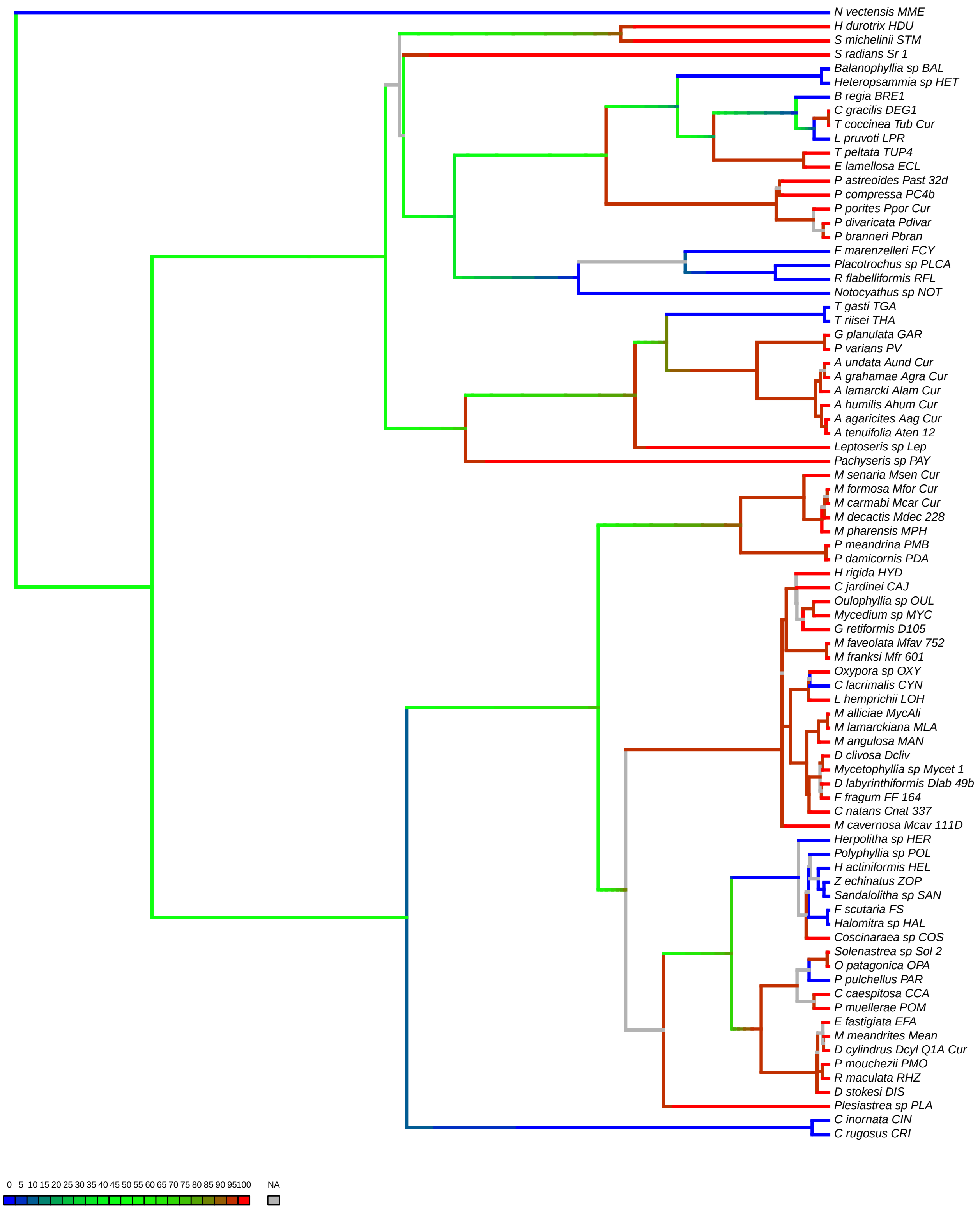


```
##
## Phylogenetic tree with 81 tips and 80 internal nodes.
##
## Tip labels:
##  A_lamarcki_Alam_Cur, H_durotrix_HDU, C_jardinei_CAJ, B_regia_BRE1, L_hemprichii_LOH, A_agaricites_A
```

```
##
## Rooted; includes branch lengths.
```

It is also possible to analyse the number of transitions on each branch by changing the *type* parameter to *lmt* and specifying a transition between two state instead of just one state (comma separated). In this case the legend doesn't show a percentage, but the absolute expected value.

```
plot_distribution_tree(mpd, 'colonial,solitary', type='lmt', conf_level=95)
```

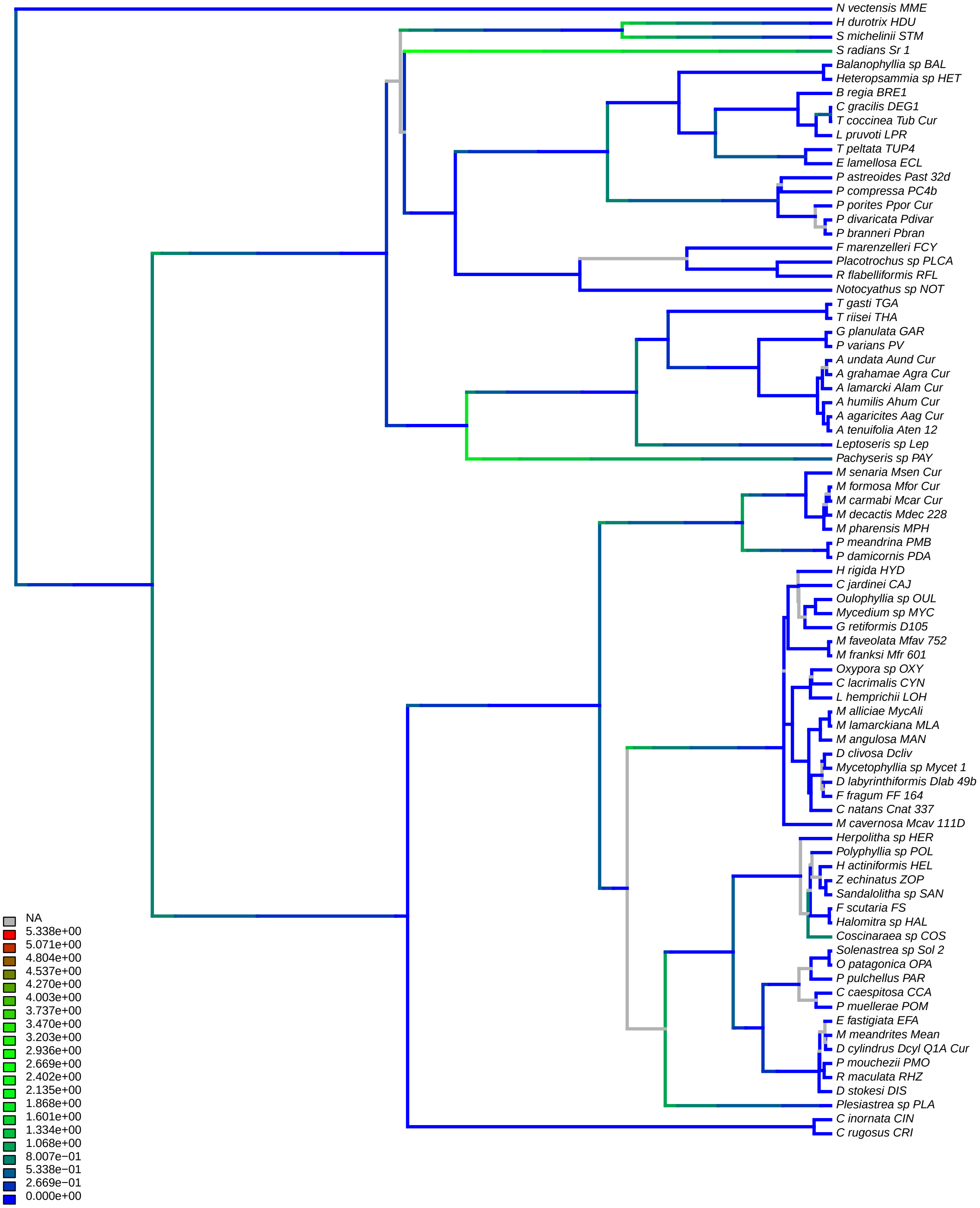


```
##
## Phylogenetic tree with 81 tips and 80 internal nodes.
##
## Tip labels:
##  A_lamarcki_Alam_Cur, H_durotrix_HDU, C_jardinei_CAJ, B_regia_BRE1, L_hemprichii_LOH, A_agaricites_A
```

```
##
## Rooted; includes branch lengths.
```

7 Correlation matrix

It is possible to plot a correlation matrix to compare results provided by different parameters on *sfreemp.map* or even different programs, as long as they return a *phylo* object with mapping on *mapped.edge*.

You can compare as many mappings as you want. Note that *correlation* supports the + operator, so you can easily accumulate results.

```
# Estimate Q using 'empirical' and 'mcmc' methods
data1 <- sfreemap(sfreemap.corals.trees[[1]], sfreemap.corals.tips, method='empirical', parallel=FALSE)
data2 <- sfreemap(sfreemap.corals.trees[[1]], sfreemap.corals.tips, method='mcmc', n_simulation=1, paral

# Now using make.simmap from the package phytools
require(phytools)
```

```
## Loading required package: phytools
```

```
## Loading required package: maps
```

```
data3 <- make.simmap(sfreemap.corals.trees[[1]], sfreemap.corals.tips, Q='mcmc', n_simulation=1)
```

```
## Done.
```

```
# Finally creating 'correlation' object
cor <- correlation(data1, 'colonial', 'sfreemap empirical') +
       correlation(data2, 'colonial', 'sfreemap mcmc') +
       correlation(data3, 'colonial', 'simmap mcmc')
```

In the *correlation* function the first parameter is the mapping, the second is the character state you want to look at, and the third one is a unique identifier.

At last, let's see the result in a nice image:

```
plot(cor)
```

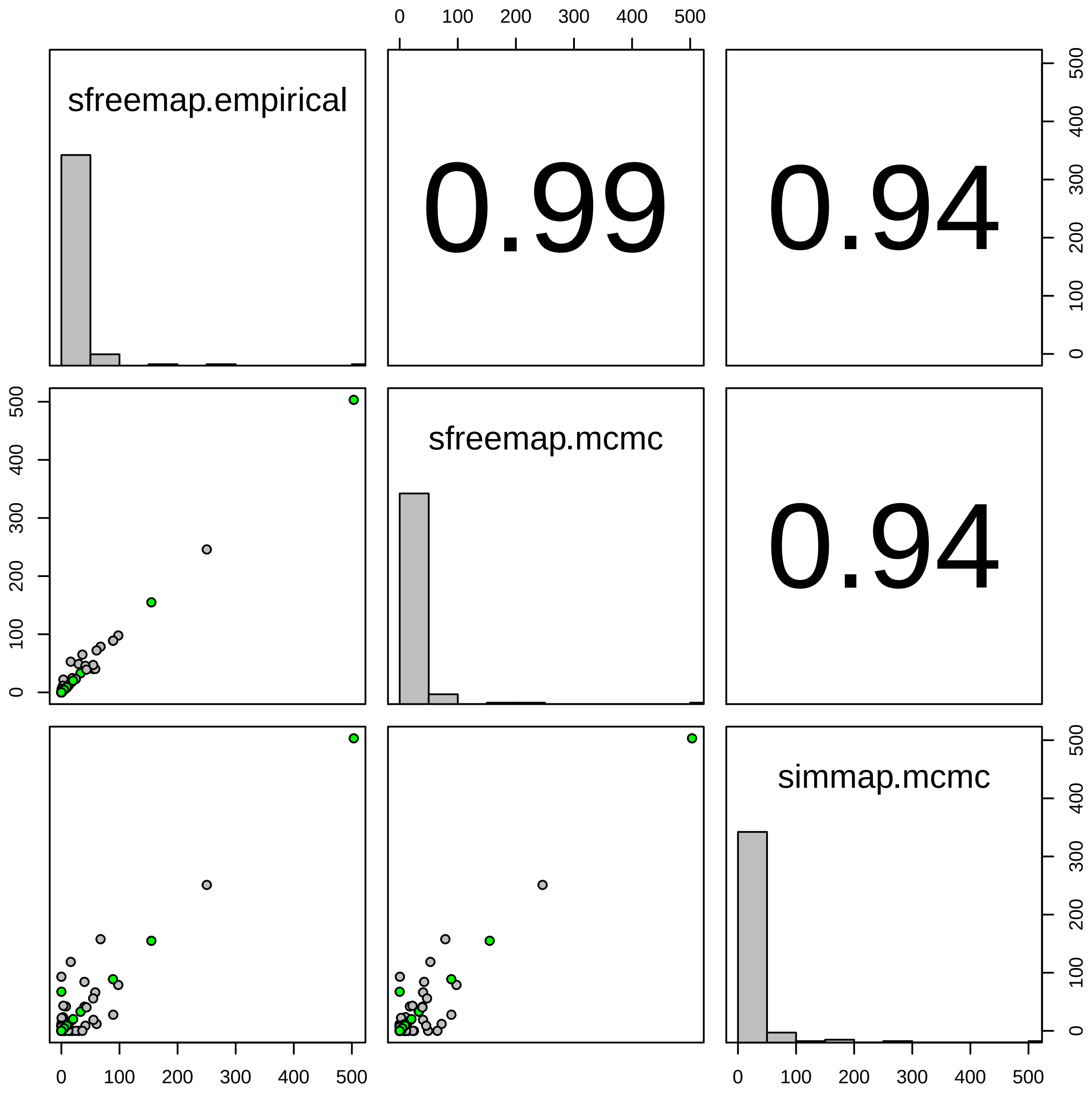
sfreemap.empirical
0.99
0.94
sfreemap.mcmc
0.94
simmap.mcmc
0
100
200
300
400
500
