# Supplementary material for: SFREEMAP - A simulation-free tool for stochastic mapping
Source: BMC Bioinformatics. 2017 Feb 22;18:123. doi: 10.1186/s12859-017-1554-7 (PMC5322606; doi:10.1186/s12859-017-1554-7)
Supplement: Additional file 2: — AF2 Ref. Manual. Sfreemap Reference manual. (PDF 119 kb) [file 12859_2017_1554_MOESM2_ESM.pdf]

Package ‘sfreemap’

January 11, 2016

Type Package

Title Simulation Free Stochastic Mapping

Version 1.0

Author Diego Pasqualin

Depends R (>= 3.1.0)

Imports Rcpp (>= 0.11.6), ape (>= 3.0-10), phytools (>= 0.3-93), reshape2(>= 1.4.1), ggplot2(>= 1.0.1), parallel (>= 3.2.1), phangorn (>= 1.99.14), seqinr (>= 3.1-3)

Suggests testthat (>= 0.10.0), knitr (>= 1.11)

Maintainer Diego Pasqualin <dpasqualin@c3sl.ufpr.br>

Description More about what it does (maybe more than one line).

License GPL (>= 2)

LazyData true

LinkingTo Rcpp, RcppArmadillo

VignetteBuilder knitr

NeedsCompilation yes

R topics documented:

sfreemap-package 2
correlation 4
describe.sfreemap 5
map_posterior_distribution 5
plot.correlation 7
plot_distribution_chart 7
plot_distribution_tree 8
pruning 10
read_tips 10
reorder.sfreemap 11
rescale 12
sfreemap 13

sfreemap.corals.tips 15
sfreemap.corals.trees 15
sfreemap.primates.dna.tips 16
sfreemap.primates.summary.tree 16
sfreemap.primates.trees 17

Index **18**

`sfreemap-package` *Simulation Free Stochastic Mapping*

Description

More about what it does (maybe more than one line).

Details

The DESCRIPTION file:

Package:	sfreemap
Type:	Package
Title:	Simulation Free Stochastic Mapping
Version:	1.0
Author:	Diego Pasqualin
Depends:	R (>= 3.1.0)
Imports:	Rcpp (>= 0.11.6), ape (>= 3.0-10), phytools (>= 0.3-93), reshape2(>= 1.4.1), ggplot2(>= 1.0.1), parallel (>
Suggests:	testthat (>= 0.10.0), knitr (>= 1.11)
Maintainer:	Diego Pasqualin <dpasqualin@c3sl.ufpr.br>
Description:	More about what it does (maybe more than one line).
License:	GPL (>= 2)
LazyData:	true
LinkingTo:	Rcpp, RcppArmadillo
VignetteBuilder:	knitr

Index of help topics:

```
correlation             Creates an object that can be used to plot a
                        correlation matrix
describe.sfreemap       describe a 'phylo' object modified by sfreemap
map_posterior_distribution
                        Analyses a set of trees regarding to a specific
                        tree and output an object that can be used by
                        many other functions of this package
plot.correlation        Plot a correlation matrix
plot_distribution_chart
                        Plot the distribution of states in a given node
plot_distribution_tree
```

	Plot the dwelling times distribution of a state in a given tree
pruning	Prune a phylogetic tree
read_tips	Read the tip states and labels from a file
reorder.sfreemap	Reorder a 'phylo' object modified by sfreemap
rescale	Rescale branches of a 'phylo' or 'multiPhylo' object to a given height
sfreemap	Simulation free stochastic character mapping on a phylogenetic tree
sfreemap-package	Simulation Free Stochastic Mapping
sfreemap.corals.tips	Tip data for sfreemap.corals.trees
sfreemap.corals.trees	Partial sequences of the 12S and 28S rRNA for 108 species of scleractinian corals and one sea anemone
sfreemap.primates.dna.tips	Tip data for sfreemap.primates.trees
sfreemap.primates.summary.tree	Taxonomic sampling of Old World monkeys
sfreemap.primates.trees	Taxonomic sampling of Old World monkeys

Further information is available in the following vignettes:

sfreemap	sfreemap (source, pdf)

This package can be used to perform a stochastic mapping in one or more phylogenetic trees, providing as result the expected number of transitions of states and the dwelling times for each branch of the tree.

Afterwards it is possible to analyse the results through charts and plots from tools provided in this package.

The method implemented is based on an article written by Vladimir Minin and Marc Suchard, describing a way to perform analytical stochastic mapping, without requiring to simulations as most methods do. As a result of this, the execution of most functions is really fast.

Author(s)

Diego Pasqualin

Maintainer: Diego Pasqualin <dpasqualin@c3sl.ufpr.br>

References

Vladimir N Minin e Marc A Suchard. Fast, accurate and simulation-free stochastic mapping. Philosophical Transactions of the Royal Society B: Biological Sciences, 363(1512):3985-3995, 2008.

`correlation` *Creates an object that can be used to plot a correlation matrix*

Description

Creates an object that can be used to plot a correlation matrix using plot.correlation.

Usage

```
correlation(map, state, name)
```

Arguments

`map`	A `sfreemap` object, result of sfreemap execution;
`state`	The character state that will be used to calculate The correlation;
`name`	A string to uniquely identify this mapping.

Details

Correlation matrix is useful to check if different methods implies on similar results. To if you run sfreemap with different parameters you can easily check the correlation of the outcomes by summing up objects created with this function and plottig it using plot.correlation.

This function returns an object that can handle the plus operator. See examples.

Value

An object of class `correlation`

Author(s)

Diego Pasqualin <dpasqualin@inf.ufpr.br>

See Also

sfreemap, plot.correlation

Examples

```
r1 <- sfreemap(sfreemap.corals.trees[[1]], sfreemap.corals.tips, parallel=FALSE)
r2 <- sfreemap(sfreemap.corals.trees[[1]], sfreemap.corals.tips, method='mcmc',
                  n_simulation=1, parallel=FALSE)

cor <- correlation(r1, 'colonial', 'empirical') + correlation(r2, 'colonial', 'mcmc')
plot(cor)
```

`describe.sfreemap` *describe a* `phylo` *object modified by sfreemap*

Description

This function returns the expected number of transitions and dwelling times of for the states of the tree passed as argument.

If tree is a `multiPhylo` the result will be the mean value for the trees.

Usage

```
describe.sfreemap(tree, ...)
```

Arguments

`tree`	A result of a sfreemap execution, can be of types `phylo` or `multiPhylo`
`...`	Other arguments

Value

A named list with values `transitions` and `dwelling_times` for each state.

Author(s)

Diego Pasqualin <dpasqualin@inf.ufpr.br>

See Also

`sfreemap`

`map_posterior_distribution` *Analyses a set of trees regarding to a specific tree and output an object that can be used by many other functions of this package*

Description

This function will compared the `trees` against the `base_tree` and return an object that can be used with plot_distribution_chart to plot the posterior distribution for dwelling times and number of transistions on a particular node and plot_distribution_tree, which plots the posterior distribution in the entire tree.

Usage

```
map_posterior_distribution(base_tree, trees, scale.branches=TRUE
                                        , scale.trees=FALSE, parallel=TRUE)
```

Arguments

- `base_tree` — A single tree, result of the function sfreemap;
- `trees` — A `multiPhylo` object, result of sfreemap execution
- `scale.branches` — Whether the function should scale the dwelling times to the branch length, resulting in a proportion of the time instead of an absolute value (the expected number of transitions is not scaled).
- `scale.trees` — A value representing the maximum height to scale the tree, or FALSE if the trees should not be scaled.
- `parallel` — Whether the function should run in parallel (defaults to TRUE)

Value

A named list with three items:

- `emr` for expected markov reward (the dwelling times for states) and `lmt` for the labelled markov transitions (the expected number of state transitions).

  Each criteria (emr and lmt) is composed by an array three dimentions, like this: $emr[trees, states, nodes]$.

  This structure represent the corresponding value for each node and state on each tree in `trees` when compared o `base_tree`. When a node from `base_tree` has no match, the corresponding state values will be set to NA.
- `base_tree` the original base_tree given as argument. This is useful for the functions that use the result of this function, cited in the description.

Author(s)

Diego Pasqualin <dpasqualin@inf.ufpr.br>

See Also

sfreemap

Examples

```
sm <- sfreemap(sfreemap.corals.trees, sfreemap.corals.tips, parallel=FALSE)
map <- map_posterior_distribution(sm[[1]], sm, parallel=FALSE)
p <- plot_distribution_chart(map, 160)
print(p)
```

plot.correlation — *Plot a correlation matrix*

Description

Plot a correlation matrix.

Usage

```
## S3 method for class 'correlation'
plot(x, y=NULL, ...)
```

Arguments

x	A result of correlation function;
y	Not used;
...	Not used.

Details

Plot obj, result of correlation.

Author(s)

Diego Pasqualin <dpasqualin@inf.ufpr.br>

See Also

correlation

plot_distribution_chart — *Plot the distribution of states in a given node*

Description

This function plots the posterior distribution for a state (or all states) in particular node, highlighting the confidence interval desired calculated using the highest posterior density (HPD).

Usage

```
plot_distribution_chart(map, nodes=NULL, trees=NULL, states=NULL, conf_level=95
                        , number_of_ticks=20, type='emr')
```

Arguments

- `map` A result of a map_posterior_distribution execution;
- `nodes` A vector containing the nodes to filter, or NULL to not filter;
- `trees` A vector containing the trees to filter, or NULL to not filter;
- `states` A vector containing the states to filter, or NULL to not filter;
- `conf_level` An integer representing the confidence level desired, ranging from 0 to 100. Defaults to 95 percent;
- `number_of_ticks` The number of intervals in the x axis. Defaults to 20;
- `type` `emr` for expected markov reward (dwelling times) or `lmt` for labelled markov transitions (number of transitions). Defaults to `emr`;

Value

This function returns a ggplot object that shows the plot when printed. The print itself can be changed as the pleased, using regular ggplot layers functions.

Author(s)

Diego Pasqualin <dpasqualin@inf.ufpr.br>

See Also

`map_posterior_distribution`, `sfreemap`

Examples

```
sm <- sfreemap(sfreemap.corals.trees, sfreemap.corals.tips, parallel=FALSE)
map <- map_posterior_distribution(sm[[1]], sm, parallel=FALSE)
p <- plot_distribution_chart(map, 160)
print(p)
```

---

plot_distribution_tree

Plot the dwelling times distribution of a state in a given tree

---

Description

This functions plots the posterior distribution of a state in the tree used as base_tree on map_posterior_distribution.

The legend shows the colors representing the incidence of `state` given as parameter in the trees analysize in `map`. The NA values means that there are no sufficient data to compute the values for the branch given the confidence interval (`conf_level` parameter). In other words, the branch is not present in a sufficient number of the analysed trees.

Usage

```
plot_distribution_tree(map, state, type='emr', conf_level=95, number_of_ticks=20
                        , tip_states=NULL, fsize=0.7, ftype="i", lwd=3)
```

Arguments

- `map` — A result of a map_posterior_distribution execution;
- `state` — The state to be plotted
- `type` — `emr` for expected markov reward (dwelling times) or `lmt` for labelled makov transistions (number of transitions). Defaults to `emr`;
- `conf_level` — An integer representing the confidence level desired, ranging from 0 to 100. Defaults to 95 percent;
- `number_of_ticks` — The number of intervals in which the data will be divided. Defaults to 20;
- `tip_states` — tip states as provided to `sfreemap`;
- `fsize` — relative font size for tip labels;
- `ftype` — font type - options are "reg", "i" (italics), "b" (bold), or "bi" (bold-italics);
- `lwd` — line width for three branches.

Value

Returns the tree plotted, with the data used in the plot stored in `tree$maps`.

Author(s)

Diego Pasqualin <dpasqualin@inf.ufpr.br>

See Also

`map_posterior_distribution`

Examples

```
sm <- sfreemap(sfreemap.corals.trees, sfreemap.corals.tips, parallel=FALSE)
map <- map_posterior_distribution(sm[[1]], sm, parallel=FALSE)
tree <- plot_distribution_tree(map, state='colonial', tip_states=sfreemap.corals.tips)
```

pruning — *Prune a phylogetic tree*

Description

Returns t1 with only the tips that are in t2 too. Optionally reroot t1.

Usage

```
pruning(t1, t2, reroot=NULL)
```

Arguments

t1	The tree to be prunned (phylo object.
t2	The base tree (phylo object.)
reroot	Optional. The node that should represent the new root of t2

Value

A phylo object representing the prunned tree.

Author(s)

Diego Pasqualin <dpasqualin@inf.ufpr.br>

read_tips — *Read the tip states and labels from a file*

Description

Read a CSV file given as argument and return a matrix representing the states of the tips and its labels (taxa names).

The CSV file must be in a specific format. The first column contain the taxa names, each one in a different row. Every subsequent column is a character, and the state of the character is specified by each taxa on each row, forming a matrix.

You can have as many characters (columns) as you want, but you must specify one to be read, using the character argument, which is an integer representing the column of the character (first character is in column 1).

When some taxon have an ambiguous state, put all states side-by-side, without an separator. For instance, if taxa X can be at both states 'a' and 'b', write 'ab' in the file.

Usage

```
read_tips(file, character=1, sep="\t")
```

Arguments

`file`	The path to the file containing the data.
`character`	If the file contains more than one character, you can specify which one to read by the column index.
`sep`	The csv separator, defaults to TAB.

Details

If you decide to use spaces to separate taxa names from characters make sure the taxa names doesn't have spaces. For instance: "A_grahamae_Agra_Cur" is correct, "A grahamae Agra Cur" is wrong.

Value

A matrix with the taxa label as the row names and a number of columns equal to the number of possible states of the character. The values of the matrix can be one, when the taxon can be at the state, and 0 otherwize.

Author(s)

Diego Pasqualin <dpasqualin@inf.ufpr.br>

`reorder.sfreemap` *Reorder a* `phylo` *object modified by sfreemap*

Description

This function reorders the edges (and all related objects) of an object `phylo` modified by sfreemap.

From `reorder.phylo` (package ape):

In the "cladewise" order each clade is formed by a series of contiguous rows. In the "postorder" order, the rows are arranged so that computations following pruning-like algorithm the tree (or postorder tree traversal) can be done by descending along these rows (conversely, a preorder tree traversal can be performed by moving from the last to the first row). The "pruningwise" order is an alternative "pruning" order which is actually a bottom-up traversal order (Valiente 2002). (This third choice might be removed in the future as it merely duplicates the second one which is more efficient.) The possible multichotomies and branch lengths are preserved.

Usage

```
## S3 method for class 'sfreemap'
reorder(x, ...)
```

Arguments

x	A tree, result of a sfreemap execution;
...	• order: The resulting order. Can be `cladewise` or `pruningwise`.<br>• index.only: logical value indicating whether only an index should be returned.<br>• other arguments, passed to reorder.phylo

Value

A phylogenetic tree of class `phylo` with its edges reordered.

Author(s)

Diego Pasqualin <dpasqualin@inf.ufpr.br>

See Also

`sfreemap`, `reorder.phylo`

`rescale` *Rescale branches of a* `phylo` *or* `multiPhylo` *object to a given height*

Description

Rescale a `phylo` or `multiPhylo` object to a given height.

Usage

```
rescale(tree, height=NULL, parallel=FALSE)
```

Arguments

tree	One or more trees, result of a sfreemap execution;
height	The desired height to scale branches to.
parallel	Whether computation should run in parallel. Only useful for `multiPhylo`

Value

A phylogenetic tree of class `phylo` or `multiPhylo` with its branches rescaled.

Author(s)

Diego Pasqualin <dpasqualin@inf.ufpr.br>

See Also

`sfreemap`

sfreemap — *Simulation free stochastic character mapping on a phylogenetic tree*

Description

This function performs an analitic stochastic character mapping on a phylogenetic tree (algorithym proposed by Minin and Suchard).

It can be called with a combination of parameters, much like any vectorized function in R. In other words, calling it with `N` trees (`multiPhylo` object) and a single rate matrix `Q` will return `N` mapped trees. Calling `sfreemap` with a single tree (`phylo` object) and `M` `Q` matrices will result in `M` mapped trees, replicas of the single tree with the algorithm applied to it using all `Q` matrices. Same logic applies to `prior` as well. It is important to note though that it you pass on `N > 1` trees and `M > 1` rate matrices (or priors), `M` and `N` should be equal.

Usage

```
sfreemap(tree, tip_states, Q=NULL, type="standard", model="SYM"
    , method="empirical", ...)
```

Arguments

tree — a phylogenetic tree as an object of class `"phylo"`, or a list of trees as an object of class `"multiPhylo"`.

tip_states — Two formats are accepted:

- A named vector containing the states of the nodes at the tips as values, and the taxa labels as names;
- A matrix with characters as columns, tip labels as rows and the state as values.

Q — The transition rate matrix. Can be given as a matrix with state names on dimensons or estimated by the program. Options for estimation depend on argumentos `method`, `model` and `type`.

type — The type of the `tip_states` being analysed. It can be "standard", usually used for morphological characters, or "dna", for nucleotideos. Default to "standard".

model — By choosing to estimate Q user can then select a model.

When `type="standard"` the available methods are:

- `"SYM" (default)`: symmetrical model, e.g, `matric(c(0,1,2,1,0,3,2,3,0))`;
- `"ER"`: equal rates model, for example `matrix(c(0,1,1,0))`;
- `"ARD"`: all rates different model, for example `matrix(c(0,1,2,0))`;

When `type="dna"` the available methods are JC, F81, K80, HKY, TrNe, TrN, TPM1, K81, TPM1u, TPM2, TPM2u, TPM3, TPM3u, TIM1e, TIM1, TIM2e, TIM2, TIM3e, TIM3, TVMe, TVM, SYM and GTR.

method — The `method` argument is only used when `type='standard'` and the available options are:

- `empirical (default)`: first it fits a continuous-time reversible Markov model for the evolution of x and then simulates stochastic character histories using that model and the tip states on the tree. This is the same procedure that is described in Bollback (2006), except that simulation is performed using a fixed value of the transition matrix, Q, instead of by sampling Q from its posterior distribution (phytools).
- `"mcmc"`: samples n_simulation Q matrices from the posterior probability distribution of Q using MCMC, then performs stochastic maps conditioned on each sampled value of Q.

`...` Optional parameters, listed below:

- `"prior"`: the prior distribution on the root node of the tree. Options are:
  - `"equal"` (default): root node is sampled from the conditional scaled likelihood distribution at the root;
  - `"estimated"`: the stationary distribution is estimated by numerically solving `pi*Q=0`;
- `"tol"` (default: 1e-8): the tolerance for zero elements in Q, elements less then tol will be set to tol;
- `"parallel"` (default: TRUE): when `tree` is of type `multiPhylo` we can run sfreemap in parallel. The number of processes created will be the same as the cores available in your machine.
- When `Q="mcmc"` some other parameters might be set:
  - `"n_simulations"` (default: 100): The number of Q matrices that will be generated;
  - `"burn_in"` (default: 1000): the burn in for the MCMC;
  - `"sample_freq"` (default: 100): number of generations for each sample taken;

Value

Returns a modified object of class "phylo", adding the following data:

`mapped.edge` a matrix containing the expected value for dwelling times for each state along each edge of the tree;

`mapped.edge.lmt`
a matrix containing the expected number of labelled markov transitions for each state along each edge of the tree;

`Q` the given or estimated value of Q;

`logL` The likelihood of calculated for the given or sampled Q;

`prior` The prios given or calculated for the root node;

Author(s)

Diego Pasqualin <dpasqualin@inf.ufpr.br>

References

Vladimir N Minin e Marc A Suchard. Fast, accurate and simulation-free stochastic mapping. Philosophical Transactions of the Royal Society B: Biological Sciences, 363 (1512): 3985-3995, 2008.

`sfreemap.corals.tips` *Tip data for sfreemap.corals.trees*

Description

One binary character with states "solitary" "colonial"). Complete description on sfreemap.corals.trees

Usage

```
sfreemap.corals.tips
```

Format

A named vector containing the states as values and species as names

References

Barbeitos, Marcos S., Sandra L. Romano, and Howard R. Lasker. "Repeated Loss of Coloniality and Symbiosis in Scleractinian Corals." Proceedings of the National Academy of Sciences 107, no. 26 (2010): 11877-82.

`sfreemap.corals.trees` *Partial sequences of the 12S and 28S rRNA for 108 species of scleractinian corals and one sea anemone*

Description

This dataset comprises partial sequences of the 12S and 28S rRNA for 108 species of scleractinian corals and one sea anemone (used as outgroup) and one binary character (0 = solitary and 1 = colonial species). The posterior distribution of trees was sampled via MCMC using the CIPRES Science Gateway (http://www.phylo.org/sub_sections/portal/) implementation of BEAST (Drumond et al., 2007). Sequences were aligned with reference to a consensus secondary structure model (details in Barbeitos et al., 2010) and data were partitoned into loop and stems. Analyses were run under the Hasegwa Kishino Yano substitution model (HKY, Hasegawa et al., 1985) with rate heterogeneity among sites modeled by a gamma distribution discretized into 4 categories and a relaxed (uncorrelated) lognormal molecular clock. The chain was run for 10 million generations and sampled every 1,000 generations. Stationarity was assessed using Tracer (Rambaut et al., 2014) after the first 10% chain was discarded. In order to maximize independence among samples, the ensuing distribution of trees was "thinned" to 901 trees, i.e, sub-sampled until the integrated autocorrelated time (IACT) dropped to 0 (see Pagel et al., 2006 for details).

Usage

```
sfreemap.corals.trees
```

Format

A "multiPhylo" object containing 901 phylogenetic trees

References

Barbeitos, Marcos S., Sandra L. Romano, and Howard R. Lasker. "Repeated Loss of Coloniality and Symbiosis in Scleractinian Corals." Proceedings of the National Academy of Sciences 107, no. 26 (2010): 11877-82.

Drummond, Alexei, and Andrew Rambaut. "BEAST: Bayesian Evolutionary Analysis by Sampling Trees." BMC Evolutionary Biology 7 (2007): 214.

Hasegawa, M., H. Kishino, and T. Yano. "Dating the Human-ape Split by a Molecular Clock of Mitochondrial DNA." Journal of Molecular Evolution 22 (1985): 160-74.

`sfreemap.primates.dna.tips` *Tip data for sfreemap.primates.trees*

Description

Complete description on sfreemap.primates.trees

Usage

```
sfreemap.primates.dna.tips
```

Format

A matrix containing the species as row names and characters as columns

---

`sfreemap.primates.summary.tree` *Taxonomic sampling of Old World monkeys*

Description

Summary of sfreemap.primates.trees

Usage

```
sfreemap.primates.summary.tree
```

Format

A "phylo" object

`sfreemap.primates.trees`
Taxonomic sampling of Old World monkeys

Description

Taxonomic sampling of Old World monkeys (parvorder Catarrhini, which includes humans, gorillas, chimpanzees, orangutans, gibbons, baboons, macaques and langurs) used by Pagel et al. (2006), plus one New World monkey (the tufted capuchin, Cebus apella), which was used as outgroup. Complete and partial cytochrome b sequences were downloaded from GeneBank and aligned using the online version of MAFFT (Katoh et al., 2007). The alignment was converted to amino acid sequences and trimmed so that the reading frame starts at the first site. Data were partitioned by codon position and analyzed under the general-time reversible (GTR, Tavare, 1986) substitution model with rate heterogeneity among sites also modeled by a 4-category gamma distribution and rate variation across branches accommodated by a relaxed (uncorrelated) lognormal molecular clock. Four independent chains were run for 100 million generation and sampled every 100,000 generations. Stationarity and convergence among chains were assessed using Tracer (after a 10% burnin) and the posterior distributions of trees were also "thinned". These posteriors were subsequently combined using LogCombiner (Drummond 2006) into a single distribution of 3,600 trees.

Usage

```
sfreemap.primates.trees
```

Format

A "multiPhylo" object containing 3596 phylogenetic trees

References

Barbeitos, Marcos S., Sandra L. Romano, and Howard R. Lasker. "Repeated Loss of Coloniality and Symbiosis in Scleractinian primates." Proceedings of the National Academy of Sciences 107, no. 26 (2010): 11877-82.

Pagel, Mark, and Andrew Meade. "Bayesian Analysis of Correlated Evolution of Discrete Characters by Reversible-Jump Markov Chain Monte Carlo." Am Nat 167, no. 6 (2006): 808-25.

Katoh, Kazutaka, Kei-ichi Kuma, Hiroyuki Toh, and Takashi Miyata. "MAFFT Version 5: Improvement in Accuracy of Multiple Sequence Alignment." Nucleic Acids Research 33, no. 2 (2005): 511-18. doi:10.1093/nar/gki198.

Tavare, S. "Some Probabilistic and Statistical Problems in the Analysis of DNA Sequences." American Mathematical Society: Lectures on Mathematics in the Life Sciences 17 (1986): 57-86.

Drummond, Alexei, and Andrew Rambaut. "BEAST: Bayesian Evolutionary Analysis by Sampling Trees." BMC Evolutionary Biology 7 (2007): 214.

Index

∗Topic **datasets**
sfreemap.corals.tips, 15
sfreemap.corals.trees, 15
sfreemap.primates.dna.tips, 16
sfreemap.primates.summary.tree, 16
sfreemap.primates.trees, 17
∗Topic **package**
sfreemap-package, 2
∗Topic **phylogenetics**
map_posterior_distribution, 5
plot_distribution_chart, 7
plot_distribution_tree, 8
reorder.sfreemap, 11
rescale, 12
sfreemap, 13
∗Topic **utilities**
correlation, 4
describe.sfreemap, 5
plot.correlation, 7
pruning, 10
read_tips, 10

correlation, 4, *7*

describe.sfreemap, 5

ggplot, *8*

map_posterior_distribution, 5, *8, 9*

phytools, *14*
plot.correlation, *4*, 7
plot_distribution_chart, *5*, 7
plot_distribution_tree, *5*, 8
pruning, 10

read_tips, 10
reorder.phylo, *11, 12*
reorder.sfreemap, 11
rescale, 12

sfreemap, *4–6*, *8*, *9*, *11*, *12*, 13
sfreemap *(*sfreemap-package*)*, 2
sfreemap-package, 2
sfreemap.corals.tips, 15
sfreemap.corals.trees, *15*, 15
sfreemap.primates.dna.tips, 16
sfreemap.primates.summary.tree, 16
sfreemap.primates.trees, *16*, 17
